# Development and evaluation of a “simulator-based” ultrasound training program for university teaching in obstetrics and gynecology–the prospective GynSim study

**DOI:** 10.3389/fmed.2024.1371141

**Published:** 2024-04-24

**Authors:** Johannes Weimer, Florian Recker, Annette Hasenburg, Holger Buggenhagen, Karla Karbach, Lia Beer, Andreas Weimer, Lina Schiestl, Liv Lorenz, Roman Kloeckner, Anna Dionysopoulou

**Affiliations:** ^1^Rudolf Frey Learning Clinic, University Medical Center of the Johannes Gutenberg University Mainz, Mainz, Germany; ^2^Department of Obstetrics and Prenatal Medicine, University Hospital Bonn, Bonn, Germany; ^3^Department of Obstetrics and Gynecology, University Medical Center of the Johannes Gutenberg University Mainz, Mainz, Germany; ^4^Center of Orthopedics, Trauma Surgery, and Spinal Cord Injury, University Hospital Heidelberg, Heidelberg, Germany; ^5^Department of Radiation Oncology and Radiotherapy, University Medical Center of the Johannes Gutenberg University Mainz, Mainz, Germany; ^6^Institute of Interventional Radiology, University Hospital Schleswig-Holstein, Lübeck, Germany

**Keywords:** ultrasound training, simulator-based training, education, obstetrics, gynecology

## Abstract

**Introduction:**

This study addresses the challenges of ultrasound education in obstetrics and gynecology, focusing on the potential benefits of simulation techniques in medical training. Aiming to evaluate the impact of a structured simulator-based training program, this prospective, randomized, interventional study examines its effects on educational outcomes for 5^th^ year medical students.

**Methods:**

A total of 153 medical students were randomized into two groups: one receiving both theoretical instruction and hands-on ultrasound simulator training (study group), and the other receiving only theoretical instruction (control group). The study assessed theoretical knowledge and practical skills at two time points: upon enrollment and at the end of the course. The practical skills were specifically evaluated using a dedicated test on the ultrasound simulator.

**Results:**

Out of 153 students, 113 completed the study (study group n=59, control group n=54). The students in the study group demonstrated a greater improvement in theoretical test scores. They also achieved better results at the practical test, with regard to image quality, accuracy, and efficiency. Both groups showed an increase in self-confidence and competency in performing ultrasound examinations independently. Students expressed high satisfaction with the course and a positive attitude toward simulator-based training.

**Discussion:**

Simulator-based training presents a valuable supplement to traditional clinical education methods in obstetrics and gynecology. This approach is particularly effective in overcoming the challenges posed by the sensitive nature of gynecological examinations in medical student training. The study highlights the benefits of integrating simulator-based methods into medical curricula, improving both theoretical and practical ultrasound skills among students.

## 1 Introduction

Ultrasound imaging, particularly transabdominal and transvaginal, is a critical diagnostic tool in Obstetrics and Gynecology (Ob/Gyn). Its non-invasive nature and excellent safety profile make it indispensable in clinical practice for differentiating between normal and pathological findings (1–3).

Gaining ultrasound imaging proficiency requires extensive training. Key medical bodies, including the EFSUMB and WFUMB, emphasize integrating ultrasound education into medical curricula (4, 5).

Traditionally, ultrasound training for medical students is conducted in clinical settings, supervised by experienced physicians. This method, though valuable, is fraught with challenges. It is time-consuming, requiring physicians to juggle their clinical responsibilities with teaching duties (6). Additionally, the patient-centered nature of this training can be stressful for students, especially during sensitive procedures like transvaginal sonography, and uncomfortable for patients who may be hesitant to be examined by inexperienced learners (7–9).

Theoretical ultrasound knowledge is gained from materials and courses, yet practical skills require hands-on practice. Present Ob/Gyn ultrasound training often fails to meet these needs, resulting in a practical skills gap among medical students (10). To address these challenges, peer-assisted learning has been proposed as an alternative. This approach involves students learning from and practicing with fellow students who have been trained as peer tutors (11, 12). While effective in certain areas like echocardiography or abdominal sonography, the intimate nature of Ob/Gyn examinations poses unique challenges for implementing peer-assisted learning with live models (13–15).

As a result, there is a growing consensus on the need for reform in ultrasound education in Ob/Gyn. This reform should align with the evolving needs of students and advancements in modern technology (16–19). Incorporating modern teaching materials and methods is crucial for developing practical specialist skills and understanding aspects of patient safety (4, 5, 20). In this regard, ultrasound simulators have emerged as a key component of innovative training concepts. They offer a risk-free environment for students to practice and hone their skills without the pressures and limitations of real patient interactions (21–23).

The use of simulation techniques in medical education is not new. Simulators have long been used for teaching anatomy, physiology, surgical techniques, and obstetric skills (24–26). Recent advancements in technology have led to the development of high-fidelity obstetric/gynecologic ultrasound simulators and even mobile ultrasound simulation applications for smartphones and tablets, enabling remote learning (27, 28).

Our study investigates how structured ultrasound training affects medical students' learning outcomes in Obstetrics/Gynecology (Ob/Gyn). It is a prospective, randomized study that assesses a program combining theory with simulator-based practice. The goal is to show that this approach significantly improves ultrasound skills in Ob/Gyn, measured by theoretical and practical simulator tests. We also look at secondary outcomes like increased self-confidence in performing real patient ultrasounds, satisfaction with the training, and interest in adopting this method in the curriculum. The results could significantly impact Ob/Gyn medical education, producing more skilled and confident practitioners.

## 2 Materials and methods

**Figure 1:**
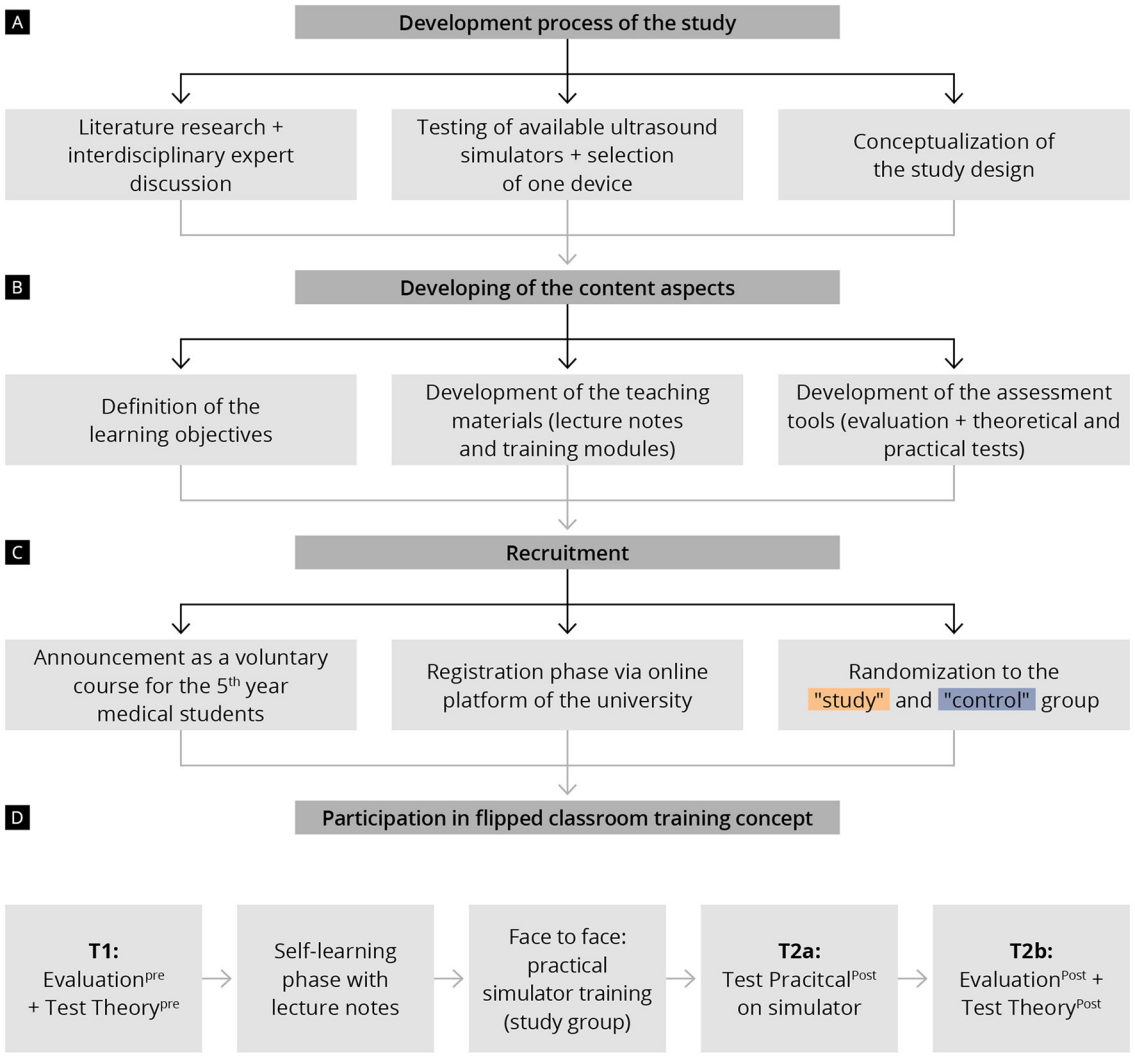
Chronological presentation of the study procedure and training course program, including data collection times (T1, T2a, and T2b) **(A)** Development process of the study; **(B)** Developing of the content aspects; **(C)** Recruitment; **(D)** Flipped classroom training concept.

This is a prospective, single-center, randomized, interventional study (Figure 1). The study was designed in accordance with the CONSORT guidelines for reporting parallel group randomized trials and under the guidance for reporting intervention development studies in health research (GUIDED) (29, 30). The study was conducted from January 2022 to December 2022. It involved 5th year medical students that were randomized 1:1 to either receive a combination of theoretical teaching and hands-on training using an ultrasound simulator (study group) or to receive the theoretical lectures alone (control group). The contents of the course program were designed after taking into consideration the quality requirements for ultrasound examination in early and second-trimester pregnancy and the updated recommendations for the performance of basic gynecologic ultrasound examinations of the German Society for Ultrasound in Medicine (DEGUM) (1–3). The training sessions and evaluation tests took place at the Department of Obstetrics and Gynecology of the University Medical Center of the Johannes Gutenberg University Mainz, Germany. The study was designed in cooperation with the Rudolf-Frey Learning Clinic of the Johannes Gutenberg University Mainz and approved by the Ethics Committee of the Medical Association of Rhineland-Palatinate (Number: 2022-16372).

### 2.1 Selection and description of the ultrasound simulator

**Figure 2:**
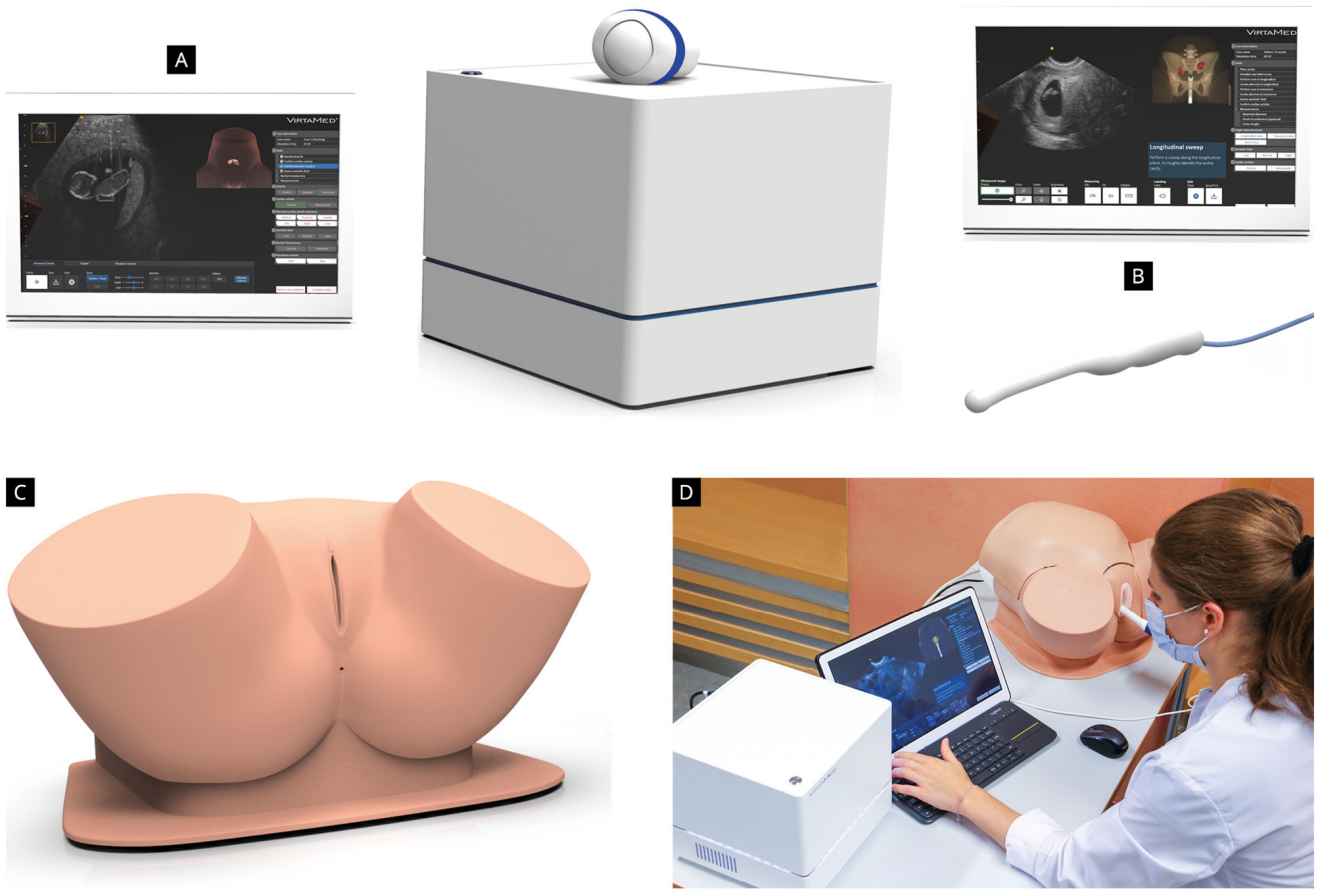
VirtaMed Portable GynoSTM™ OB/GYN ultrasound simulator (Zurich, Switzerland) consisting of an anatomic pelvic model equipped **(C, D)** with an anatomical uterus insert, two abdominal models (for fetuses younger than 18 weeks and for fetuses older than 18 weeks), as well as a transabdominal **(A)** and a transvaginal ultrasound **(B)** transducer replica.

Prior to the study, we conducted a testing phase to determine which ultrasound simulator would best meet the needs of our study population. Therefore, we invited several ultrasound simulator companies to allow our medical team to test and evaluate their products. Three of them responded and agreed to take part to the this testing phase (Scantrainer 8:TAS/TVS OBGYN-Education pack), Skillsmed (Nuremberg, Germany), VirtaMed Portable GynoS™ OB/GYN ultrasound simulator (Zurich, Switzerland) and VIM-003 (Ob/Gyn) Simulator Base Unit, CAE Vimedix (Sarasota, United States). The three ultrasound simulators were installed in the Department of Obstetrics and Gynecology of the University Medical Center of the Johannes Gutenberg University Mainz and were available for testing for five working days. Twenty-six participants (14 medical students, nine residents, and three consultants in Obstetrics and Gynecology) took part in this testing phase. The technical characteristics and functions of the simulators, the training platform and courses included in the software, as well as aspects like self-learning and simulator feedback were evaluated. We decided to conduct our study using the Virtamed Portable GynoS™ OB/GYN ultrasound simulator, since our colleagues' evaluation showed a superiority of this simulator with regard to the above-mentioned aspects (Figure 2). The simulator includes transabdominal and transvaginal obstetric ultrasound modules with more than 100 cases available in the training platform. For an overview of all possible modules of the simulator, see Supplementary material S1.

### 2.2 Participant recruitment and eligibility criteria

The study was a voluntary part of the officially predetermined curriculum of the University Medical Center of the Johannes Gutenberg University Mainz, Germany. Being a 5th year medical student, consenting to participate in the study and completing the assessment tools (evaluation forms, theoretical test, and practical test) were defined as inclusion criteria. An invitation to participate describing the scope of the study was sent via email to all 5th year medical students of winter and summer semesters 2022. Informed written consent was obtained from all participating students.

### 2.3 Course program

The course program (Figure 1), based on the flipped classroom model, consisted of a structured preparation phase via lecture notes and one dedicated onsite course with face-to-face teaching on the ultrasound simulator (only for the study group).

#### 2.3.1 Lecture notes

Both groups (study and control group) received lecture notes providing theoretical knowledge about the standard planes of a basic gynecologic and obstetric ultrasound examination (see Supplementary material S2). With the help of anatomical figures and images from real transabdominal and transvaginal ultrasound examinations, the students were guided through the most important normal and pathologic findings of Obstetrics and Gynecology. The following aspects of normal anatomy were covered: uterus in sagittal and transverse view, measurement of the endometrial thickness, normal appearance of the ovaries and pouch of Douglas, the appearance of a normal early pregnancy along with the measurement of the crown-rump length, basic biometrical measurements of the fetus in the second and third trimester, as well as the appearance and localization of the placenta and the evaluation of the cervical length and amniotic fluid volume. With regard to pathologic findings, the following topics were discussed: position variants of the uterus, ovarian cysts, early pregnancy loss, ectopic pregnancy and pregnancy of unknown location, as well as polyhydramnios, oligohydramnios and cervical shortening.

#### 2.3.2 The onsite training course

**Figure 3:**
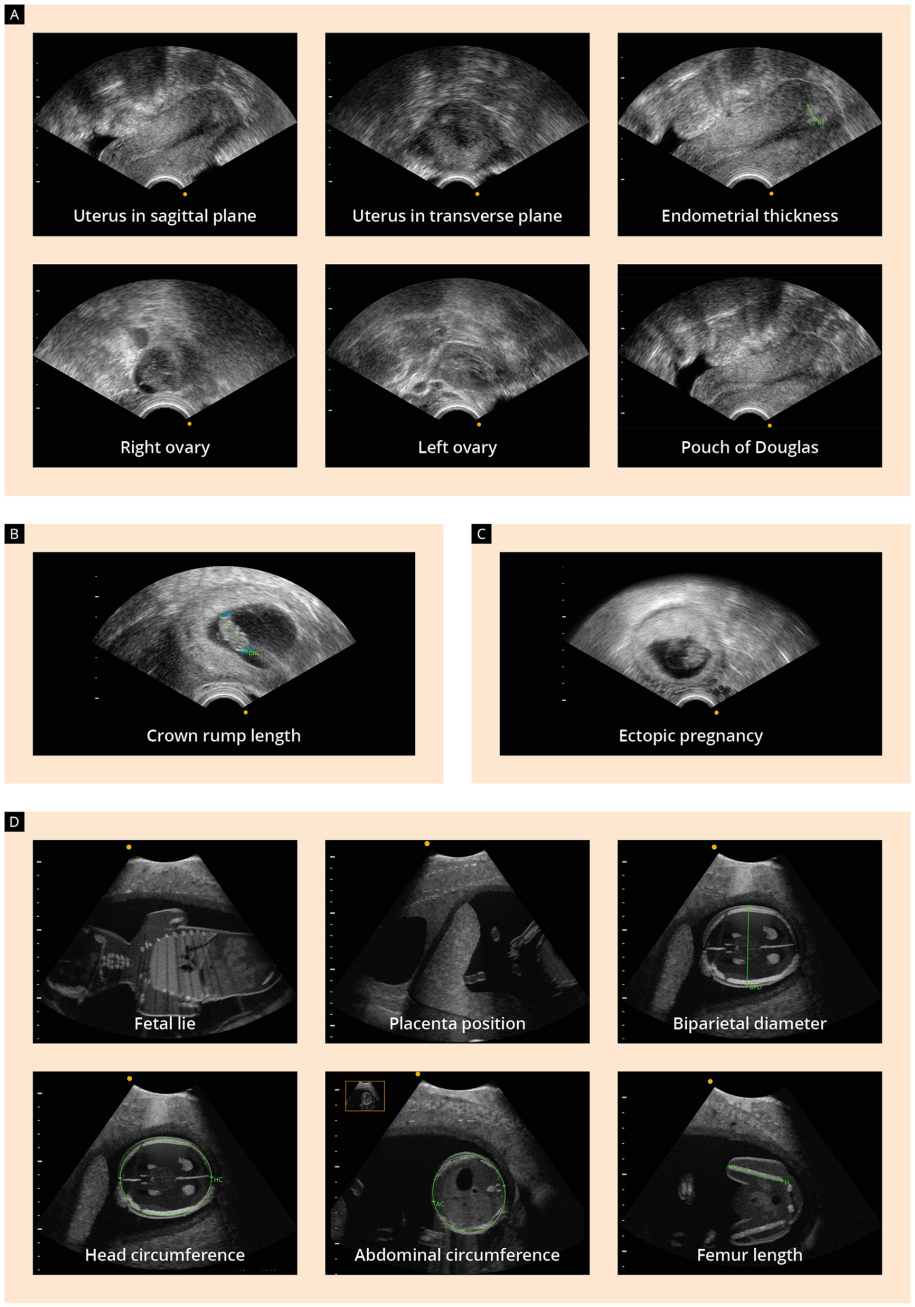
Presentation of the four modules of the onsite training course and learning objectives: **(A)** assessment of the uterus and endometrium on the sagittal and transverse plane, measurement of the endometrial thickness, identification of the ovaries and the pouch of Douglas; **(B)** identification of the embryo and measurement of the crown-rump length (CRL) on the 10th week of gestation; **(C)** visualization of an ectopic pregnancy; **(D)** second-trimester ultrasound examination, including identification of the fetal lie and placenta position and performing fetal biometric measurements.

Only students in the study group received the onsite training course on the ultrasound simulator. The control group received the aforementioned lecture notes only. All practical training sessions were identical and were performed by the same investigator after herself receiving dedicated training courses on the ultrasound simulator until she reached an expert level on the predefined training modules. The training course consisted of four modules and lasted about 75–90 min per student. The predefined learning objectives and practical tasks of each module are shown in Figure 3.

### 2.4 Evaluation forms and learning assessment

In order to measure obstetric/gynecologic ultrasound competency acquisition and the students' attitude toward the course program, written evaluations and assessment of theoretical knowledge were performed at two time points: upon enrollment (T1: Evaluation^pre^ and Test Theory^pre^) and at the end of the course program (T2b: Evaluation^post^ and Test Theory^post^). Assessment of practical skills was performed in form of a practical test on the ultrasound simulator also at the end of the course program (T2a: Test Practical^post^). All evaluation forms and learning assessment tools were designed by the authors of the study based on published assessment methods used to measure ultrasound competency in medical ultrasound education (31, 32).

#### 2.4.1 Evaluation forms at time points T1 and T2b

The demographic characteristics of the participants as well as previous experience with ultrasound on real patients and/or on simulated settings were assessed upon enrollment (time point T1). The participants' subjective level of competency in transabdominal and transvaginal sonography, their level of self-confidence with regard to ultrasound examinations on real patients, their learning goals and motivation to practice on the simulated environment were assessed at time points T1 and T2b using questions based on a 7-point Likert scale (1 = strongly disagree with the statement; 7 = strongly agree with the statement). At time point T2b (Evaluation^post^) the following aspects were also evaluated: the lecture notes, the properties of the ultrasound simulator, as well as the advantages, future perspectives and attitudes toward simulator-based ultrasound training in the field of obstetrics and gynecology.

#### 2.4.2 Learning assessment: theoretical tests at time points T1 und T2b

Theoretical knowledge related to obstetric/gynecologic ultrasound was assessed at time points T1 (Test Theory^pre^) and T2b (Test Theory^post^). The contents of the theoretical tests were based on the predefined aforementioned learning goals. Each test included 21 single choice, multiple choice and free text questions (33) that were subdivided in the following four topics: (1) uterus and Douglas Pouch, (2) ovaries and ovarian pathology, (3) early pregnancy, (4) fetus and placenta (see Supplementary material S3). For the evaluation of the results of the theoretical tests a scoring system was developed. Correct answers scored 1 point and wrong answers zero points. We did not use negative scoring for wrong answers.

#### 2.4.3 Learning assessment: test practical^post^ on the ultrasound simulator at time point T2a

In order to evaluate practical ultrasound skills, the participants underwent the Test Practical^post^ on the ultrasound simulator at time point T2a. Both groups performed the practical test independently, after receiving a short introduction into the settings and functions of the simulator. The practical test was supervised by medical experts who had previously received training on the ultrasound simulator. Technical assistance was provided whenever needed, but no feedback or instructions were given. The Test Practical^post^ (about 25 min) consisted of three cases, that were available within the simulator training platform (see Supplementary material S4).

The entire practical test was recorded for each participant. All images acquired were rated on a later time point by ultrasound experts qualitatively and quantitatively using predetermined rating criteria.

a) Qualitative assessment: The image quality was assessed. Outcome measures included the correct identification of the uterus, endometrium and adnexa, the visualization of a viable first-trimester pregnancy, the correct assessment of the fetal lie and heartbeat in the second trimester of pregnancy, the localization of the placenta and the establishment of the correct diagnosis for modules (a) and (b), using a pass/fail performance level (0: fail, 1: pass).
b) Quantitative assessment: The quantitative assessment was applied only if the students were able to identify and demonstrate correctly the required anatomical level and structure. The deviation of the students‘ following measurements from the reference value was calculated: measurement of the endometrial thickness, measurement of the crown rump length in the first trimester of pregnancy and biometric measurements (biparietal diameter, head circumference, abdominal circumference and femur length) of the fetus in the second trimester of pregnancy. The time needed to complete each module was also evaluated.

### 2.5 Statistic

In order to calculate the sample size required to detect a statistical significant effect, a power analysis was performed. Based on an expected effect size of 0.6, a significance level of 0.05 and a desired power of 0.80, the calculated sample size was set at 90 participants. The evaluations and theoretical tests were conducted digitally through an online questionnaire tool and were exported as an Excel spreadsheet. The results of the practical tests were reported manually in an Excel file. All data were manually evaluated using Microsoft Excel before analysis in R studio (RStudio Team [2020]. RStudio: Integrated Development for R. RStudio, PBC, http://www.rstudio.com, last accessed 06 01 2024) with R 4.0.3 (A Language and Environment for Statistical Computing, R Foundation for Statistical Computing, http://www.R-project.org; last accessed 06 01 2024). Binary and categorical baseline parameters are expressed as absolute numbers and percentages. Continuous data are expressed as median and interquartile range (IQR) or as mean and standard deviation (SD). Categorical parameters were compared using Fisher's exact test and continuous parameters using the Mann-Whitney test. The results of the theory test were given as a percentage. In addition, pairwise correlations of metric variables were obtained, and the correlation effect sizes and significances were calculated. Furthermore, Mann-Whitney tests were constructed to compare the influence of individual factors on the results of the theoretical and practical tests. Finally, a multivariate linear regression model was produced to compare the influence of individual factors. *P* < 0.05 were considered statistically significant.

## 3 Results

### 3.1 Participants

**Figure 4:**
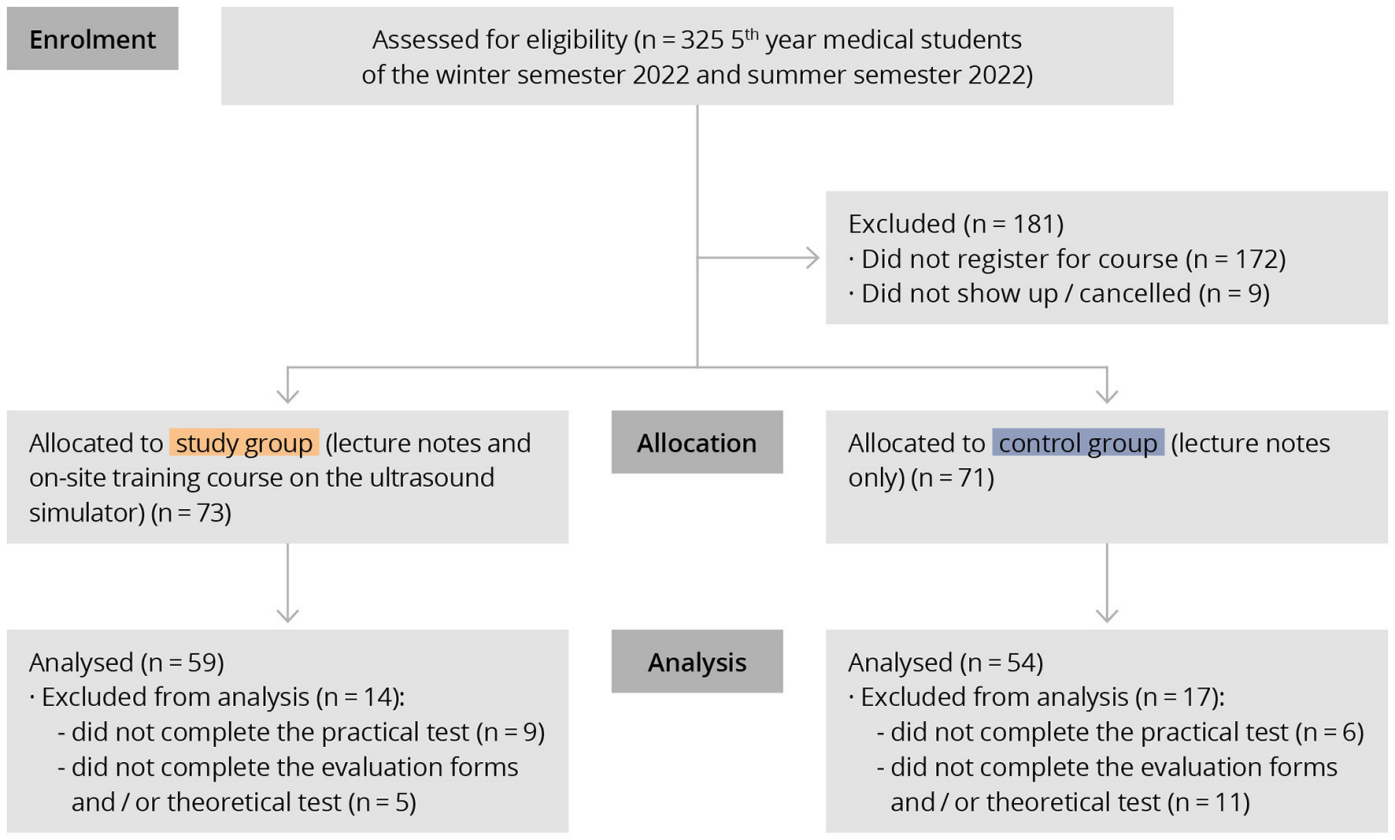
Flow Diagram showing participant recruitment and data analysis according to CONSORT guidelines.

A total of 325 5th year medical students of the winter and summer semesters 2022 were invited to participate in the study. In total, *n* = 153 students registered for the study. Nine of them canceled the course because of illness and other personal reasons. The final analysis included 113 students (study group *n* = 59, control group *n* = 54) who completed all assessment tools (Figure 4).

Participants' baseline characteristics are presented in Supplementary Table S5. There were no significant differences between the groups regarding age (study: 27.7 ± 3.3 years vs. control: 27.5 ± 4.1 years; *p* = 0.70) and the gender distribution. The majority of the students in both groups reported having previous experience with ultrasound examinations in general (study: 96.6% vs. control: 100%; *p* = 0.52), but most of them never have had contact with ultrasound simulators (study: 98.3% vs. control: 98.1%; *p* = 1.00). The only differences between the groups were that the participants in the control group stated that they had independently performed slightly more transvaginal ultrasound examinations that the ones in the study group and that within the study group, significantly more participants stated that they had already completed an apprenticeship (study: 66.1% vs. control: 40.7%; *p* = 0.01).

### 3.2 Subjective assessment of competency and self-confidence

Both groups were able to increase their ultrasound skills significantly (*p* < 0.01) over the course period. However, the study group achieved a significantly higher increase in the subjective level of skills competence (study: Δ 1.7 ± 1.0 vs. control: Δ 1.2 ±1.1; *p* = 0.03). Corresponding to that the level of self-confidence in performing ultrasound examinations independently increased to a larger extent in the study group over the study period (study: Δ 4.4 ±1.5 vs. control: Δ 3.3 ±1.5; *p* < 0.001). For further information, see Supplementary material S6.

### 3.3 Advantages of simulator-based ultrasound training, motivation and learning goals

Both groups rated the” Advantages of simulator-based ultrasound training” with high scale points (T1: study: 6.2 ± 0.80 vs. control: 6.0 ± 0.80; *p* = 0.09; T2b: study: 6.03 ± 0.81 vs. control: 5.7 ± 0.92; p = 0.07. At the end of the course, both groups rated the theme complexes “Motivation” and Learning goals” at a similarly high level, although the study group tended to rate both topic complexes with higher scale points. For further information, see Supplementary Table S7.

### 3.4 Evaluation of the teaching materials, the properties of the ultrasound simulator and future perspectives and attitudes toward simulator-based ultrasound training

Both the lecture notes (study: 5.5 ± 1.2 vs. control: 5.4 ± 1.3; *p* = 0.55) and the properties of the ultrasound simulator (study: 6.3 ± 0.8 vs. control: 6.2 ± 0.9; *p* = 0.64) were rated with similarly high scale points from both groups. The statements of the theme complex “Future perspectives and attitudes toward simulator-based ultrasound training in Obstetrics and Gynecology” were rated with significantly higher scale points from the participants of the study group. For further information, see Supplementary Table S8.

### 3.5 Learning assessment: theoretical tests and practical test on the ultrasound simulator

#### 3.5.1 Theoretical tests: test theory^pre^ and test theory^post^

**Figure 5:**
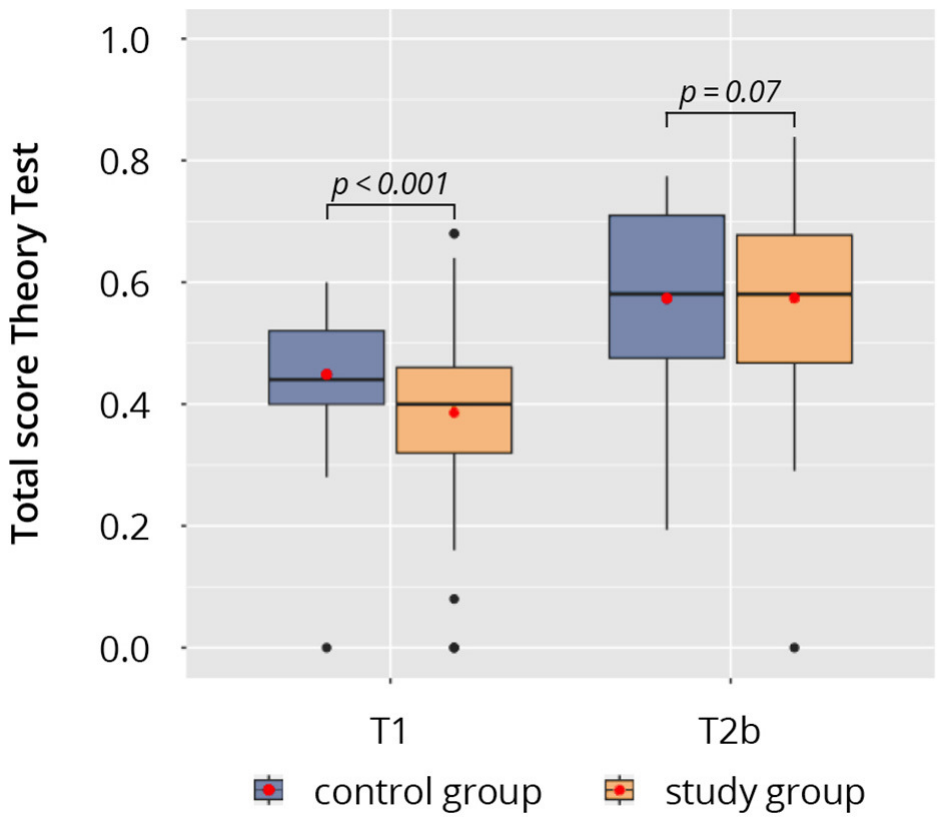
Results of the theoretical tests at time points T1 (Test Theory^pre^) and T2b (Test Theory^post^).

The results of the theoretical tests are presented in Figure 5. Upon enrollment (time point T1, Test Theory^pre^) the students in the control group achieved significantly higher scores in the theory test than the students in the study group (study: 0.39 ± 0.15 vs. control: 0.45 ± 0.10; *p* = 0.01). Both groups managed to improve their ultrasound knowledge during the course period and achieved significantly higher scores at the theoretical test at time point T2b (Test Theory^post^) (*p* < 0.001). The study group however achieved a higher increase at the total score between time points T1 and T2b (study: Δ 0.19 ± 0.17 vs. control: Δ 0.12 ± 0.17; *p* = 0.08), even though this did not reach statistical significance.

#### 3.5.2 Practical test on the ultrasound simulator (test practical^post^)

**Figure 6:**
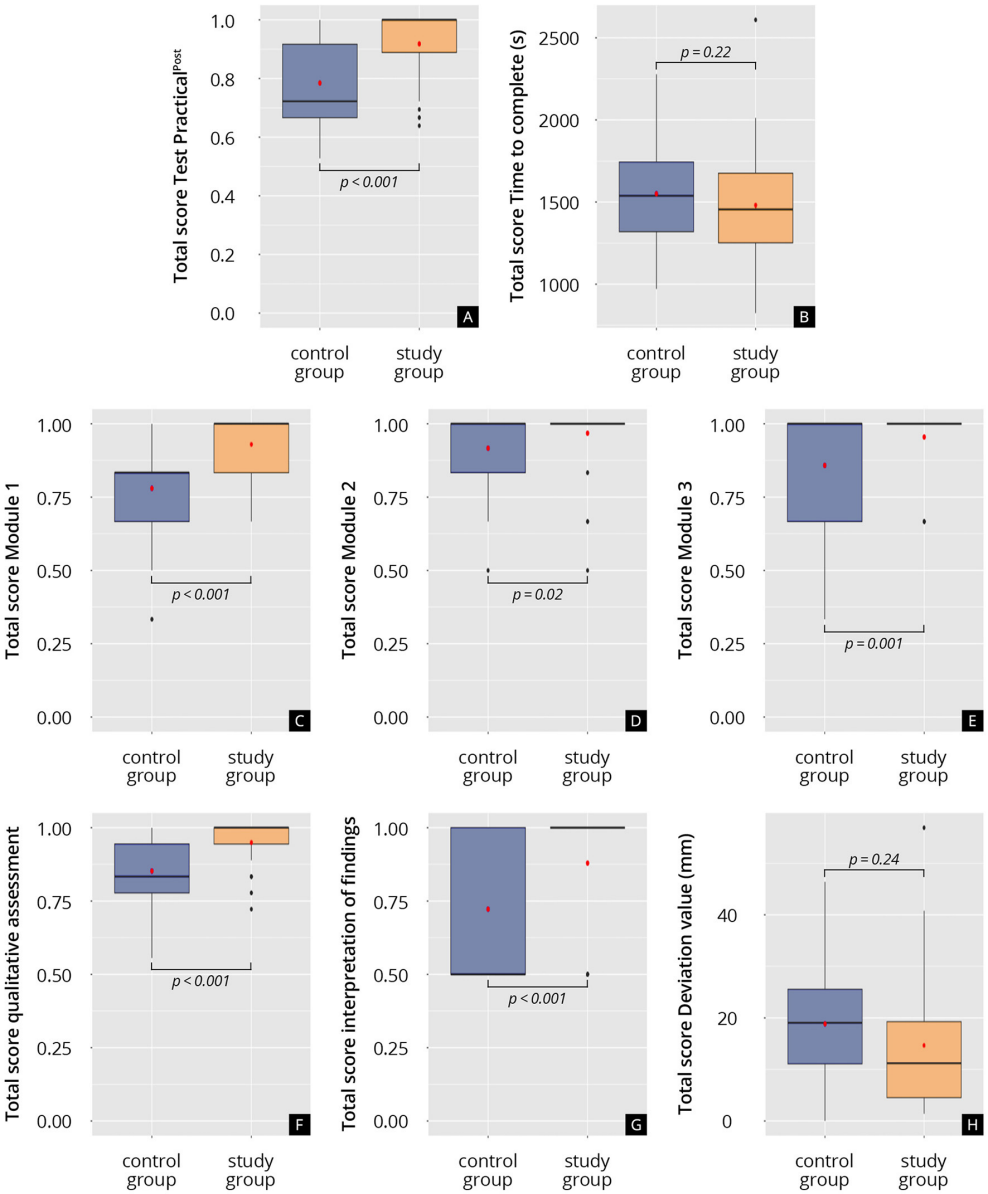
Results of the practical test on the ultrasound simulator (Test Practical^post^). **(A)** Total score on the practical test, test Practical^post^. **(B)** Time to complete the practical test, s: seconds. **(C)** Total score on Module 1 of the practical test. **(D)** Total score on Module 2 of the practical test. **(E)** Total score on Module 3 of the practical test. **(F)** Total score of the qualitative assessment analysis. **(G)** Total score of the interpretation of findings. **(H)** Total deviation of the measurements from the reference values, mm: millimeter.

The results of the practical tests on the ultrasound simulator are shown in Figure 6 and Supplementary Table S9. Qualitative and quantitative assessment was performed. Quantitative assessment was, however, only applied if the students were able to identify and demonstrate correctly the required anatomical level and structures. Because the students in the study group were significantly better at doing so (study: 0.95 ± 0.07% vs. control: 0.85 ± 0.10%; *p* < 0.001), more datasets from the study group could be taken into account in the quantitative assessment analysis. Regarding the deviation from the reference values for the measurement of the endometrial thickness, the CRL and the biometric measurements of the fetus, the study group tended to be closer to the reference values than the control group (deviation study: 14.65 ± 13.4 vs. deviation control: 18.83 ± 12.90; *p* = 0.30). In addition, the study group required on average less time to complete the tasks (study: 1,480 ± 301 s vs. control: 1,551 ± 296 s; *p* = 0.22) and was significantly better at correctly interpreting the ultrasound findings than the control group (study: 0.88 ± 0.22% vs. control: 0.72 ± 0.25%; *p* < 0.001).

### 3.6 Regression analysis and correlations

Multivariable linear regression was performed to identify influential factors and potential confounders (see Supplementary Table S10). Only the subjective level of competency in obstetric/gynecologic ultrasound at time point T1 had a significant influence on the results of the Test Theory^pre^ (β = 0.042; *p* = 0.01). The participants' reported level of self-confidence with regard to obstetric/gynecologic ultrasound examinations, correlates with better results at the Test Theory^pre^. This means that the participants, who were feeling more confident (subjective), were also better in the objective assessment of theoretical ultrasound knowledge as well.

With regard to the results of the Test Theory^post^ the variables: “total score on the Test Theory^pre^“ (β = 0.366; *p* < 0.01) and “studied the lecture notes” (β = 0.02; *p* < 0.01) were found to have significant influence on the results. Similar to the results of the regression analysis for the Test Theory^pre^, only the subjective level of competency in obstetric/gynecologic ultrasound at time point T1 had significant influence on the results of the practical test on the ultrasound simulator (β = 0.06; *p* = 0.02).

Correlation analysis between subjective and objective data are provided in Supplementary Table S11. Significant correlations (*p* < 0.05) were found between the levels of subjective and objective competencies, as well as the participants' attitude and motivation between time points T1 and T2 (T2a and T2b) with a medium/strong effect size (0.30 ≤ r ≤ 0.95).

## 4 Discussion

This prospective, single-center, randomized study offers a thorough exploration into the evolving landscape of medical education, with a specific focus on the training of obstetric and gynecologic ultrasound skills using high-fidelity simulation technology. This study highlights the critical role of ultrasound in modern medicine, particularly in obstetrics and gynecology, and adopts a scientifically rigorous method to improve medical education through innovative teaching. Its design as a prospective, randomized trial following CONSORT and GUIDED guidelines reflects a commitment to high-quality research. The use of the VirtaMed Portable GynoS™ OB/GYN ultrasound simulator demonstrates a balance between advanced technology and practical use. Findings indicate that both groups improved their ultrasound knowledge, with the study group showing greater improvement and better practical test performance, underscoring the value of extra simulator training. One could argue, that, the group that receives an additional training intervention, is expected to also perform better in the evaluation test, than the group that receives no intervention. However, this is only partially true, since the impact on skills also depends on the quality of the intervention itself. The better scores of the study group militate in favor of the quality of the training concept. Finally, both groups showed great satisfaction with the training course and rated the teaching materials and properties of the ultrasound simulator with very high scores. The students' evaluations show clearly their positive attitude toward simulator-based ultrasound medical education and their motivation to continue training on the simulated environment.

Our results are in accordance with previous published data regarding simulator-based ultrasound training for medical students. In a multicenter randomized trial, Etienne et al. examined the effect of the addition of a simulation course on the usual training in transvaginal ultrasound for medical students that were trained in an emergency gynecological unit. The course was found to be beneficial and the students were highly satisfied with the session as an initial training method (34). Similar results reported Cook *et al*. after a one-hour simulation training session in obstetric/gynecologic ultrasound for third-year medical students (35).

Several studies have examined the use of obstetric/gynecologic ultrasound simulators in training and evaluating residents, specialist doctors and medical students. Theoretical and practical simulator training as well as simulator-based trainees' evaluation techniques have been shown to be comparable with such that are patient-based (36). Moreover, simulator-based ultrasound training has been shown to be effective not only with regard to practical skills acquisition, but also in other areas of performance, like interpretation of findings, documentation and medical decision-making (37). This study's advocacy for the integration of simulator-based training in obstetrics/gynecology curricula could potentially lead to significant advancements in medical education. This perspective is supported by recent research, which suggests that the incorporation of technology-driven, interactive learning tools can enhance the educational experience and better prepare students for clinical practice (22, 38, 39).

Simulator-based medical training cannot of course replace training on real patients (40). It can, however, be used as an adjunct to traditional clinical methods of education, especially when instruction time and patient availability is limited. This is particularly important in the field of obstetrics and gynecology where the intimate character of the gynecological examination itself poses a great challenge to medical training. Simulation provides the possibility to train practical skills and acquire competencies in a safe, stress-free environment without the fear of committing errors or the risk of harming patients.

### 4.1 Strengths and limitations of the study

The study's strengths include its randomized design, consistent teaching methods, and standardized evaluation criteria, ensuring uniform training and objective grading. However, limitations include its voluntary nature, possibly attracting more ultrasound-interested students, and varying motivation levels between groups. Additionally, the study didn't assess impacts on patient safety or care quality, though previous research suggests that simulator training improves care efficiency and reduces patient discomfort and the need for repeat examinations and trainee supervision (41).

The study's focus on the immediate impacts of simulator-based training does not address the long-term retention of skills. Understanding how well these competencies are retained over time is crucial to evaluating the effectiveness of the training method. Additionally, while simulators provide a safe and controlled environment, they lack the unpredictability and complexity of real patient interactions. This may limit the preparedness of students for real-life clinical scenarios, which often involve direct patient communication and managing unexpected findings.

There is also a concern that students might become overly dependent on the simulator environment, which might not always be replicated in actual clinical settings where such technology may not be available. The high costs and limited accessibility of high-fidelity simulators further complicate the potential for widespread implementation, particularly in resource-limited settings (42). A specific cost-benefit economic analysis was not performed in our study. Even though we recognize that this is a possible limitation of the study, there are effects of simulator-based ultrasound training, like students 'preparation for clinical practice, effects on patient care and safety, as well as the satisfaction with the educational experience, that are impossible to measure with money. The ultrasound simulator is still available in our clinic for medical students to practice voluntarily and following projects are already planned.

The study predominantly focuses on quantitative assessments of knowledge and skill, possibly overlooking qualitative aspects such as learning style preferences or the subjective experience of learning with simulators. Additionally, the control group in the study received only theoretical teaching without practical hands-on experience, not considering other practical learning methods that do not involve high-fidelity simulators. This design choice might limit the scope of the study's conclusions.

### 4.2 Conclusion

The “GynSim” study marks a significant step forward in medical education for obstetrics and gynecology ultrasound training. This study shows how structured simulator-based training positively affects 5th-year medical students' educational outcomes, enhancing their theoretical knowledge, practical skills, and confidence. The findings support the effectiveness of simulation-based training, noted for its methodological strength, including randomization, a large sample size, consistent teaching, and objective evaluation. These aspects ensure the study's reliability and contribute insights into the benefits of simulator-based training, highlighting its role in preparing students for clinical practice and advocating for further research on its long-term benefits and curriculum integration to improve patient care and safety.

## Data availability statement

The raw data supporting the conclusions of this article will be made available by the authors, without undue reservation.

## Ethics statement

The studies involving humans were approved by Ethics Committee of the Medical Association of Rhineland-Palatinate. The studies were conducted in accordance with the local legislation and institutional requirements. The participants provided their written informed consent to participate in this study.

## Author contributions

JW: Conceptualization, Data curation, Formal analysis, Investigation, Methodology, Project administration, Resources, Software, Supervision, Validation, Visualization, Writing—original draft, Writing—review & editing. FR: Methodology, Validation, Writing—review & editing. AH: Conceptualization, Investigation, Resources, Supervision, Writing—review & editing. HB: Conceptualization, Investigation, Resources, Supervision, Validation, Writing—review & editing. KK: Conceptualization, Data curation, Investigation, Methodology, Writing—review & editing. LB: Data curation, Methodology, Writing—review & editing. AW: Data curation, Formal analysis, Writing—review & editing. LS: Data curation, Visualization, Writing—review & editing. LL: Data curation, Visualization, Writing—review & editing. RK: Supervision, Writing—review & editing. AD: Conceptualization, Data curation, Formal analysis, Investigation, Methodology, Project administration, Resources, Software, Supervision, Validation, Visualization, Writing—original draft, Writing—review & editing.
