# Supplementary material for: Development and evaluation of a “simulator-based” ultrasound training program for university teaching in obstetrics and gynecology–the prospective GynSim study
Source: Front Med (Lausanne). 2024 Apr 24;11:1371141. doi: 10.3389/fmed.2024.1371141 (PMC11076731; doi:10.3389/fmed.2024.1371141)
Supplement: Supplementary file 1 [file Data_Sheet_1.PDF]

S1. Module manual of the VirtaMed Portable GynoS™ OB/GYN ultrasound simulator (Zurich, Switzerland)

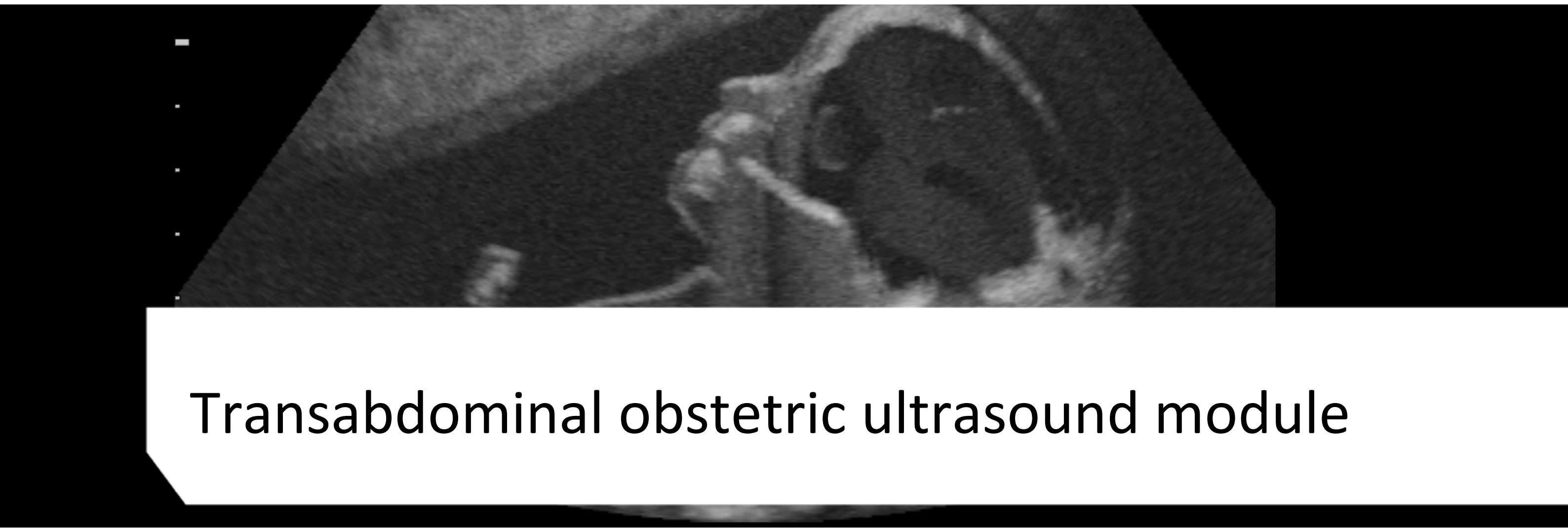

Transabdominal obstetric ultrasound module

Module description

Incorporating the 20+2 approach, a combination of 2 overview sweeps & 20 planes, the transabdominal obstetric ultrasound module provides a structured method of examining the mid-trimester fetus. Trainees learn across over 100 cases, various fetal positions, different placenta locations, and doppler imaging. The module contains various fetal abnormalities such as down syndrome, anencephaly, spina bifida, placenta previa, and bilateral renal agenesis. Training with the highest realism, the transabdominal transducer can be moved freely across the entire abdomen to visualize the fetus.

Learning objectives

- To perform a systematic second-trimester ultrasound exam using the 20+2 approach
- To gain an understanding of what the normal ultrasound appearances are in each plane
- To detect and diagnose vascular complications using doppler imaging
- To practice caliper placement for measurement of the gestational age

Instruments

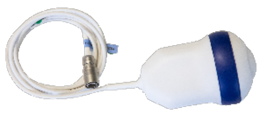

Transabdominal ultrasound transducer

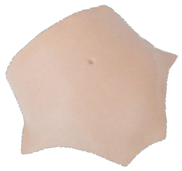

Small abdomen for fetuses younger than 18 weeks. Cases are only active when the correct abdomen is used.

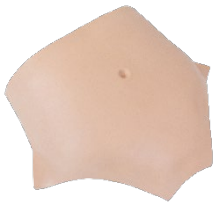

Large abdomen for fetuses older than 18 weeks. Cases are only active when the correct abdomen is used.

Transabdominal obstetric ultrasound: patients

Patient 1 “Angelique”

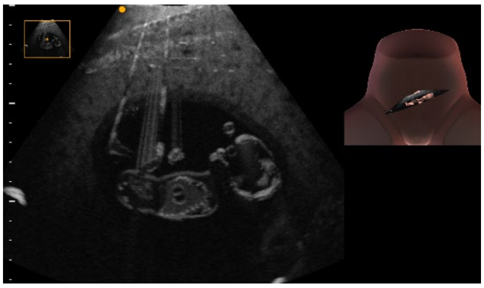

- Fetal age: 14 weeks 3 days
- Fetal position: Cephalic
- Placental location: Fundal
- Amniotic fluid: Normal
- Diagnosis: Normal pregnancy
- Belly size to use: Small
- Gender: Female

Patient 2 “Yuki”

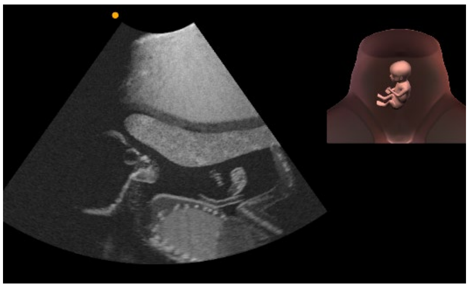

- Fetal age: 20 weeks 0 days
- Fetal position: Breech, spine left
- Placental location: Right
- Amniotic fluid: Normal
- Diagnosis: Normal pregnancy
- Belly size to use: Large
- Gender: Female

Patient 3 “Jada”

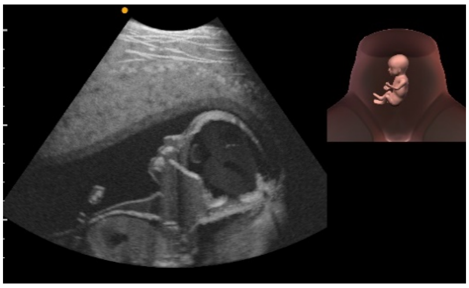

- Fetal age: 19 weeks 5 days
- Fetal position: Breech
- Placental location: Low anterior
- Amniotic fluid: Normal
- Diagnosis: Normal pregnancy
- Belly size to use: Large
- Gender: Male

Patient 4 “Ellie”

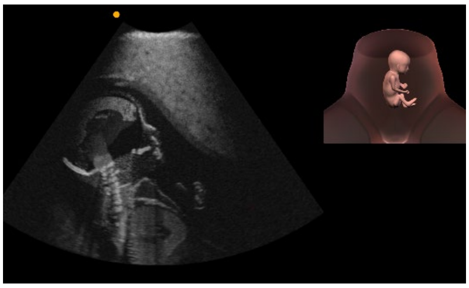

- Fetal age: 20 weeks 4 days
- Fetal position: Breech
- Placental location: Posterior
- Amniotic fluid: Normal
- Diagnosis: Normal pregnancy
- Belly size to use: Large
- Gender: Male

Patient 5 “Sofia”

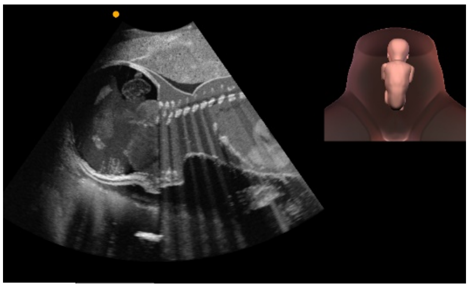

- Fetal age: 25 weeks 5 days
- Fetal position: Breech
- Placental location: Anterior fundal
- Amniotic fluid: Normal
- Diagnosis: Normal pregnancy
- Belly size to use: Large
- Gender: Female

Patient 6 "Deirdre"

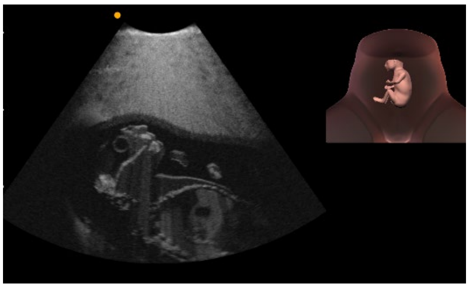

- Fetal age: 21 weeks 0 days
- Fetal position: Breech
- Placental location: Low posterior
- Amniotic fluid: Normal
- Diagnosis: Anencephaly
- Belly size to use: Large
- Gender: Male

Patient 7 "Annabelle"

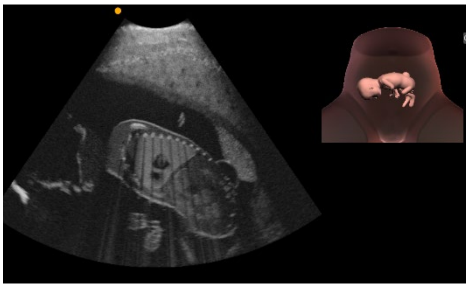

- Fetal age: 21 weeks 3 days
- Fetal position: Transverse
- Placental location: Fundal
- Amniotic fluid: Normal
- Diagnosis: Spina bifida
- Belly size to use: Large
- Gender: Male

Patient 8 "Femi"

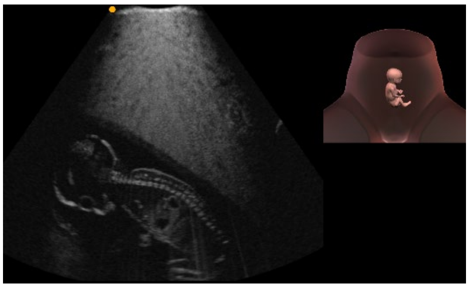

- Fetal age: 17 weeks 0 days
- Fetal position: Breech, spine right
- Placental location: Posterior fundal
- Amniotic fluid: Normal
- Diagnosis: Miscarriage
- Belly size to use: Small
- Gender: Male

Patient 9 "Priya"

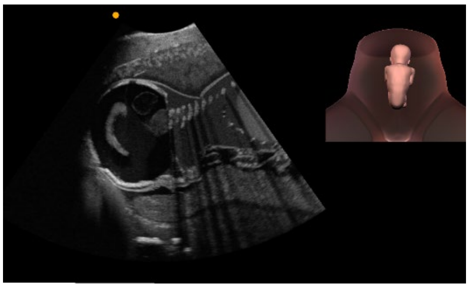

- Fetal age: 24 weeks 0 days
- Fetal position: Breech, spine up
- Placental location: Posterior
- Amniotic fluid: Low
- Diagnosis: Renal agenesis
- Belly size to use: Large
- Gender: Male

Patient 10 "Taylor"

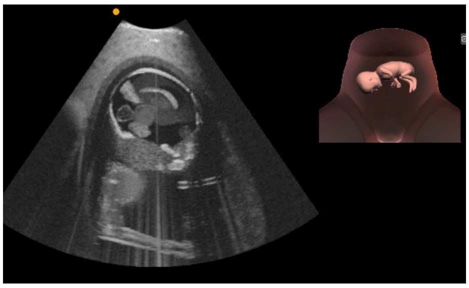

- Fetal age: 21 weeks 3 days
- Fetal position: Transverse, spine up
- Placental location: Placenta previa
- Amniotic fluid: Normal
- Diagnosis: Normal pregnancy
- Belly size to use: Large
- Gender: Female

Patient 11 "Tiara"

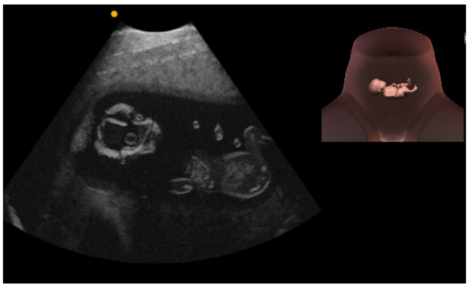

- Fetal age: 16 weeks 5 days
- Fetal position: Transverse, back
- Placental location: Fundal
- Amniotic fluid: Normal
- Diagnosis: Down syndrome
- Belly size to use: Small
- Gender: Female

Patient 12 "Ursula"

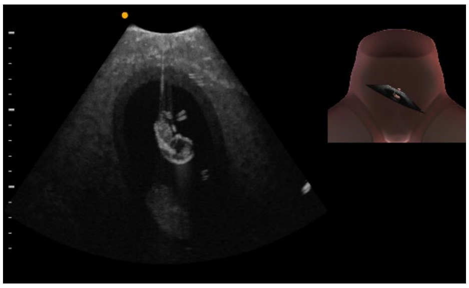

- Fetal age: 10 weeks 5 days
- Fetal position: Cephalic
- Placental location: Posterior, right
- Amniotic fluid: Normal
- Diagnosis: Normal pregnancy
- Belly size to use: Small
- Gender: Female

Patient 13 "Lucy"

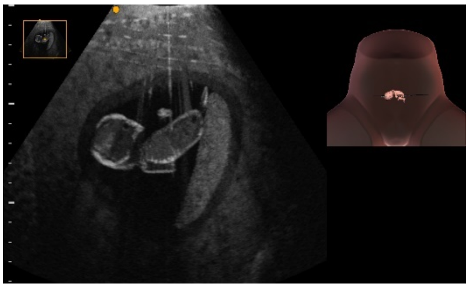

- Fetal age: 12 weeks 2 days
- Fetal position: Transverse
- Placental location: Left
- Amniotic fluid: Normal
- Diagnosis: Normal pregnancy
- Belly size to use: Small
- Gender: Male

Patient 14 "Olivia"

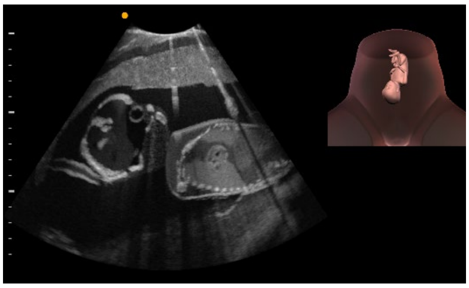

- Fetal age: 19 weeks 4 days
- Fetal position: Cephalic
- Placental location: Low lying anterior
- Amniotic fluid: Normal
- Diagnosis: Cleft lip
- Belly size to use: Large
- Gender: Female

Patient 15 "Kiki"

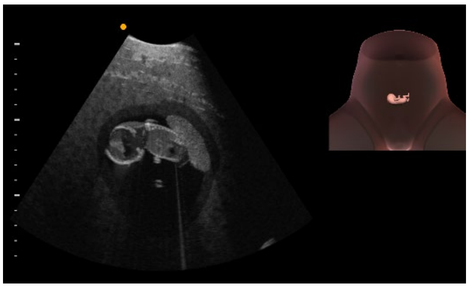

- Fetal age: 12 weeks 2 days
- Fetal position: Transverse
- Placental location: Anterior
- Amniotic fluid: Normal
- Diagnosis: Down syndrome
- Belly size to use: Small
- Gender: Male

Transabdominal obstetric ultrasound: cases

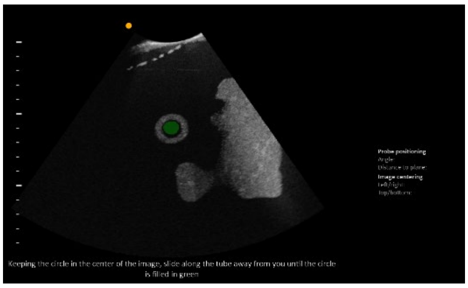

Basic Skills – Probe handling

Learning objectives:

- Slide, rotate and tilt the probe to visualize shapes
- Sweep through objects in the abdominal space to understand how probe movements affect the image on the screen

---

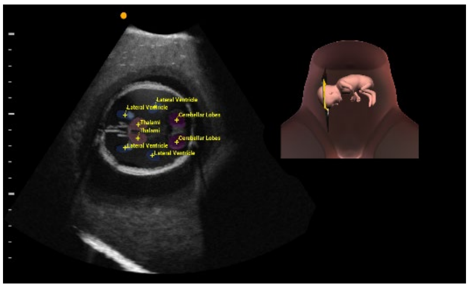

Basic Skills – Anatomical planes

Learning objectives:

- Learn the 20 important planes in the mid-trimester fetus
- Use training features such as control lights, the outside view, and anatomy labels to guide you

---

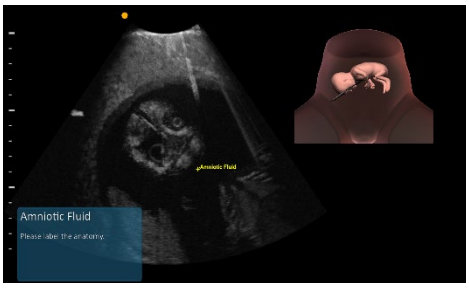

Basic Skills – Anatomies Identification

Learning objectives:

- Navigate to randomized anatomical structures and check the accuracy of your assessment

---

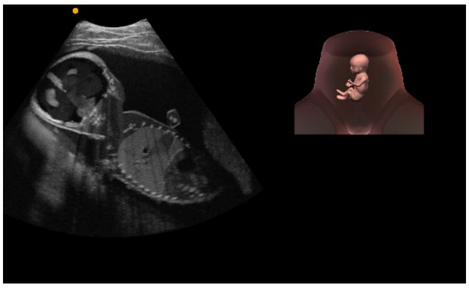

6-Steps approach

Learning objectives:

- Assess the fetal lie
- Identify placental location
- Measure amniotic fluid volume
- Visualize cardiac activity
- Perform biometric measurements based on the Hadlock scale

All training cases include one fetus only (multiple pregnancies are not simulated)

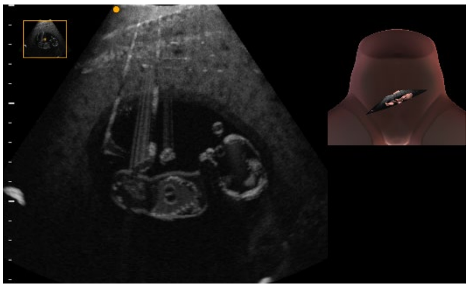

11-14 Weeks exam

Learning objectives:

- Perform the 6-steps approach exam as described above
- Measure nuchal translucency

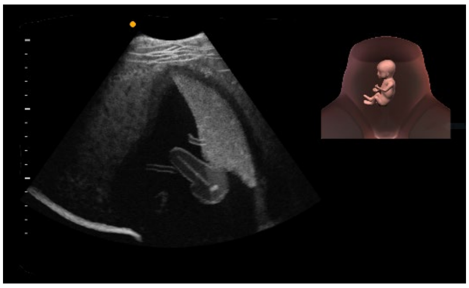

Free roam

Learning objectives:

- Navigate the different structures of the fetus according to your needs
- Recognize signs of abnormalities including placenta previa
- Perform fetal biometry

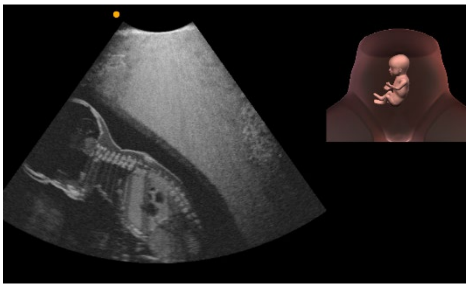

Spine

Learning objectives:

- Identify and document the 3 planes of the fetal spine: spine in sagittal, spine in coronal, and the coronal section of the body
- Check for any spinal or skin defects including spina bifida meningocele

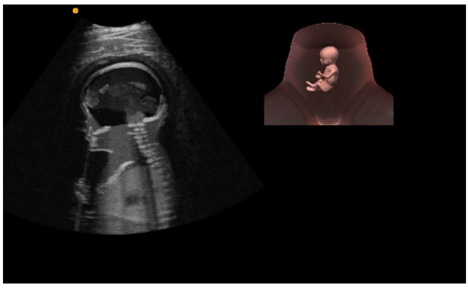

20+2 planes

Learning objectives:

- Navigate to the 20 important planes in the mid-trimester fetus
- Identify the key structures within these planes
- Recognize signs of abnormalities including bilateral renal agenesis, miscarriage, and Down syndrome
- Perform fetal biometry

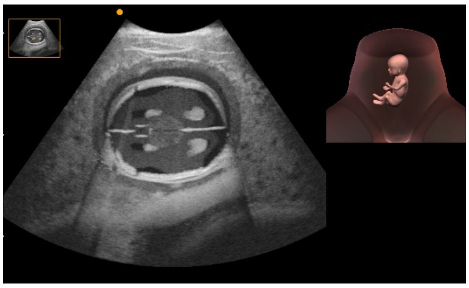

Brain

Learning objectives:

- Navigate to the 3 important planes of the brain: transventricular, transthalamic, and transcerebellar
- Identify the key structures within these planes such as the falx, ventricles, and cavum septum pellucidum
- Recognize signs of abnormalities of the brain including anencephaly, lemon-shaped scull, and banana-shaped cerebellum
- Perform fetal biometry of the brain

Heart and thorax

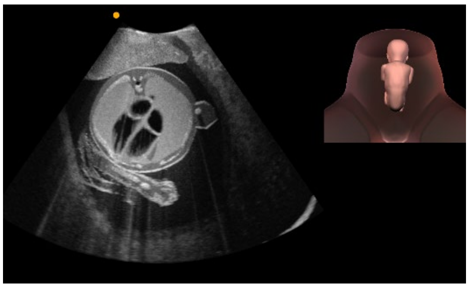

Learning objectives:

- Identify the key structures in the fetal heart such as the four-chamber view with lungs, RVOT, and LVOT
- Identify right and left side of the fetal situs
- Perform fetal biometry of the fetal heart based on the Hadlock scale
- Recognize signs of abnormalities of the heart including the ventricular septum defect

Abdomen and pelvis

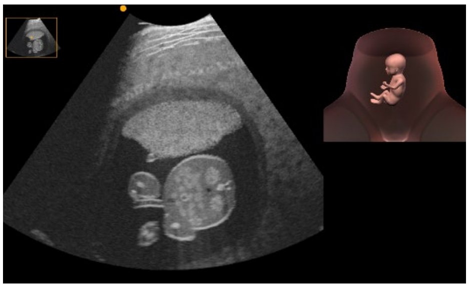

Learning objectives:

- Identify the key structures within the fetal abdomen and pelvis such as the stomach and kidneys
- Recognize signs of abnormalities of the abdomen including bilateral renal agenesis

Limbs

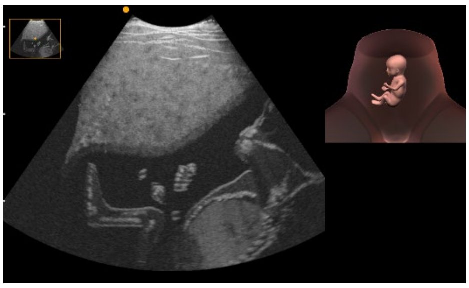

Learning objectives:

- Identify the key structures of fetal limbs such as the femur, tibia, and fibula
- Identify right and left side of the fetus
- Perform fetal biometry
- Recognize signs of abnormalities of the limbs including the sandal gap

Face

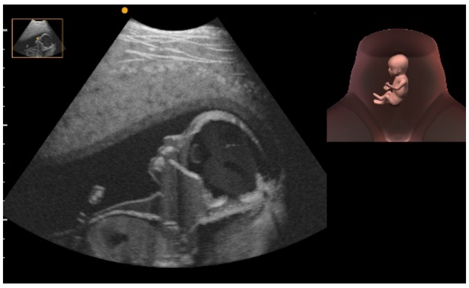

Learning objectives:

- Identify the key structures of fetal face such as the facial profile, lips, eyes, and nose
- Recognize signs of abnormalities of the face including missing nasal bone

Patient breakdown

Module	Angelique	Yuki	Jada	Ellie	Sofia	Deirdre	Annabelle
6-Steps	-	Case 9	Case 1	Case 6	Case 8	-	Case 4
11-14 Weeks	Case 3	-	-	-	-	-	-
Free Roam	Case 1	Case 2	Case 3	Case 4	Case 5	Case 6	Case 7
20+2	-	-	-	Case 3	Case 6	-	Case 4
20+2 Testing	Case 3	Case 1	Case 12	Case 7	Case 10	Case 8	Case 9
Spine	Case 8	-	-	Case 9	Case 7	Case 1	Case 2
Brain	-	Case 2	Case 1	Case 7	Case 5	-	Case 6
Heart	Case 6	Case 8	Case 3	Case 5	Case 2	Case 11	Case 9
Abdomen	-	Case 8	Case 10	Case 7	Case 1	Case 4	Case 3
Limbs	Case 10	Case 6	Case 2	Case 1	Case 4	Case 7	Case 9
Face	Case 5	Case 1	Case 4	Case 3	Case	Case 8	Case 7

Module	Femi	Priya	Taylor	Tiara	Ursula	Lucy	Olivia	Kiki
6-Steps	Case 5	Case 10	Case 7	Case 2	-	-	Case 3	
11-14 Weeks	-	-	-	-	Case 2	Case 1		Case 4
Free Roam	Case 8	Case 9	Case 10	Case 11	Case 12	Case 13	Case 14	Case 15
20+2	-	Case 5	Case 1	Case 2	-	-	-	
20+2 Testing	Case 2	Case 4	Case 11	Case 6	-	-	Case 5	
Spine	Case 6	Case 5	Case 4	Case 3	-	-	-	
Brain	Case 4	Case 9	Case 3	Case 8	-	-	-	
Heart	Case 4	Case 1	Case 10	Case 7	-	-	-	
Abdomen	Case 9	Case 6	Case 2	Case 5	-	-	-	
Limbs	Case 11	Case 8	Case 5	Case 3	-	-	-	
Face	Case 6	-	-	Case 2	-	-	-	

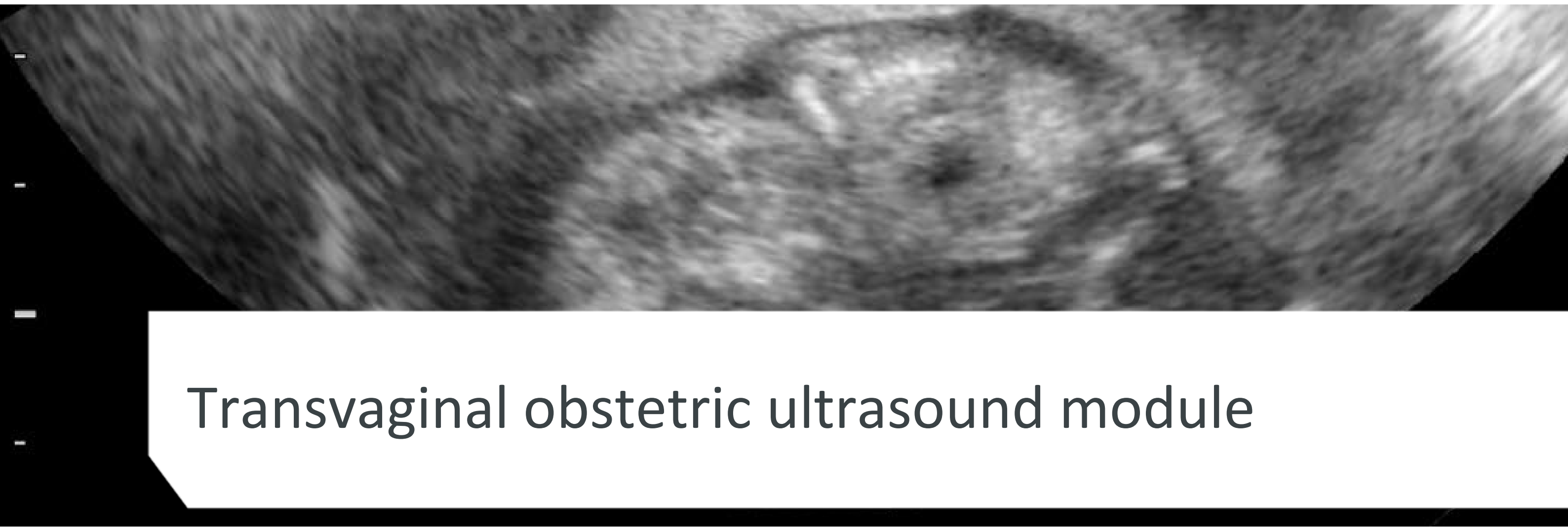

Transvaginal obstetric ultrasound module

Module description

A comprehensive training for 1st trimester transvaginal ultrasound, the module contains 12 patient cases of which 7 are abnormalities (2 early pregnancy losses, 3 pregnancies of unknown location, 1 double ectopic pregnancy, and 1 non-pregnant patient). Uterine abnormalities such as masses and fluids in the adnexa, as well as Nabothian cysts, are also included. The transfer of skills from the simulator to the patient is facilitated thanks to the realistic tactile sensation of the transvaginal probe.

Learning objectives

- To perform a systematic first-trimester ultrasound exam
- To visualize and assess uterine and pregnancy structures
- To practice caliper placement for measurement of the gestational age

Instruments

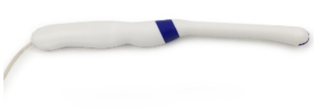

Transvaginal ultrasound probe

Transvaginal obstetric ultrasound: guided cases

Patient 1 “Chante”

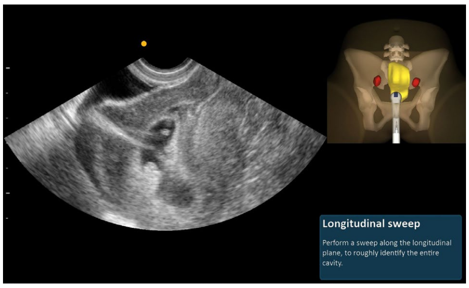

- Pregnancy classification: Early pregnancy loss
- Number of embryos: 1
- Actual gestational age: 6w, 6d
- EGA based on LMP: 6w, 6d
- Landmarks not possible to visualize: All are possible to visualize.
- Previous pregnancies: 1 Gravida, 0 Para, 0 c-section, 0 ectopic
- Previous ultrasound for current pregnancy: None
- Recent medical history: N/A

Patient 2 “Akira”

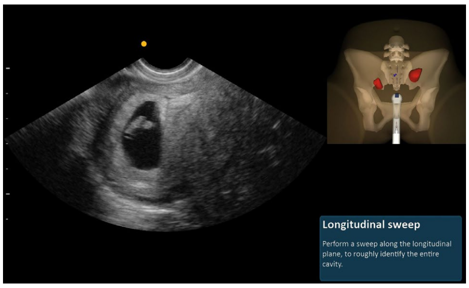

- Pregnancy classification: Definite intrauterine pregnancy
- Number of embryos: 1
- Actual gestational age: 7w, 1d
- EGA based on LMP: 6w, 3d
- Landmarks not possible to visualize: All are possible to visualize.
- Previous pregnancies: 3 Gravida, 2 Para, 0 c-section, 0 ectopic
- Previous ultrasound for current pregnancy: None
- Recent medical history: N/A

Patient 3 “Galia”

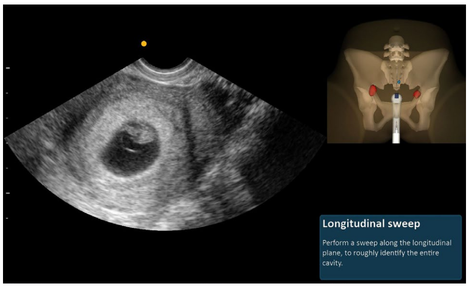

- Pregnancy classification: Definite intrauterine pregnancy
- Number of embryos: 1
- Actual gestational age: 8w, 1d
- EGA based on LMP: 8w, 1d
- Landmarks not possible to visualize: All are possible to visualize.
- Previous pregnancies: 1Gravida, 0 Para, 0 c-section, 0 ectopic
- Previous ultrasound for current pregnancy: None
- Recent medical history: N/A

Patient 4 “Jasmine”

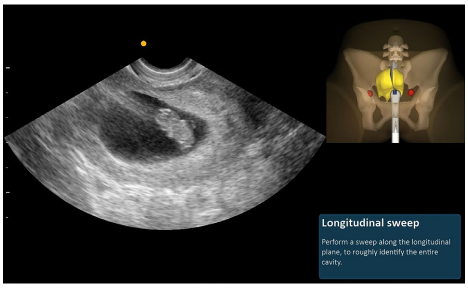

- Pregnancy classification: Definite intrauterine pregnancy
- Number of embryos: 1
- Actual gestational age: 9w, 1d
- EGA based on LMP: 9w, 1d
- Landmarks not possible to visualize: All are possible to visualize.
- Previous pregnancies: 1 Gravida, 0 Para, 0 c-section, 0 ectopic
- Previous ultrasound for current pregnancy: None
- Recent medical history: Nabothian cysts on cervix

Patient 5 “Sasha”

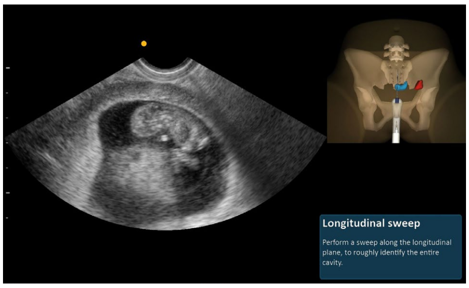

- Pregnancy classification: Definite intrauterine pregnancy
- Number of embryos: 1
- Actual gestational age: 10w
- EGA based on LMP: 13w, 2d
- Landmarks not possible to visualize: All are possible to visualize.
- Previous pregnancies: 3 Gravida, 1 Para, 1 c-section, 0 ectopic
- Previous ultrasound for current pregnancy: None
- Recent medical history: N/A

Patient 6 “Riley”

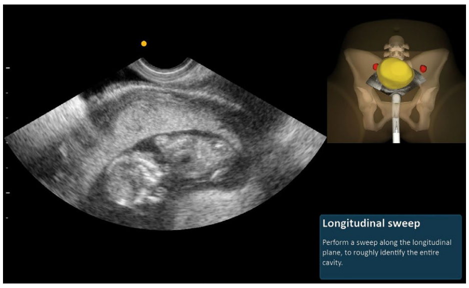

- Pregnancy classification: Definite intrauterine pregnancy
- Number of embryos: 1
- Actual gestational age: 12w, 1d
- EGA based on LMP: 11w, 5d
- Landmarks not possible to visualize: Yolk sac is not present.
- Previous pregnancies: 2 Gravida, 1 Para, 1 c-section, 0 ectopic
- Previous ultrasound for current pregnancy: None
- Recent medical history: N/A

Patient 7 “Noel”

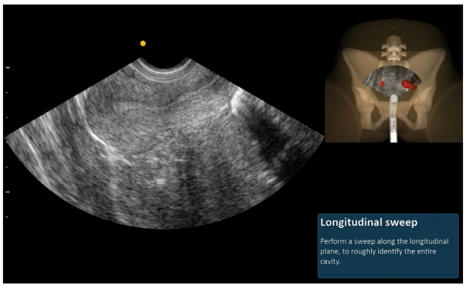

- Pregnancy classification: Pregnancy of unknown location
- Number of embryos: 0
- Actual gestational age: Unknown
- EGA based on LMP: 5w, 5d
- Notes: Pregnancy of unknown location. Patient could not be pregnant despite positive pregnancy test.
- Landmarks not possible to visualize: Gestational sac, embryo/fetus, yolk sac, and cardiac activity not present.
- Previous pregnancies: 1 Gravida, 0 Para, 0 c-section, 0 ectopic
- Previous ultrasound for current pregnancy: None
- Recent medical history: History of irregular menses, weekly positive pregnancy test

Patient 8 “Dakota”

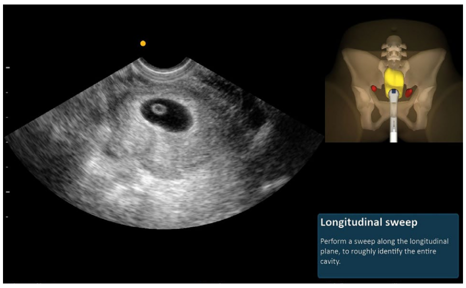

- Pregnancy classification: Early pregnancy loss (based on history only)
- Number of embryos: 0
- Actual gestational age: approx. 7w, 3d
- EGA based on LMP: 8w, 4d
- Notes: Spontaneous abortion, mean sac diameter measures at approx. 7w 3d, minimal grow since last ultrasound.
- Landmarks not possible to visualize: Embryo/fetus not present. No cardiac activity is present.
- Previous pregnancies: 2 Gravida, 1 Para, 0 c-section, 0 ectopic
- Previous ultrasound for current pregnancy: Yes, scanned 2 weeks ago at 6w, 6d
- Recent medical history: Irregular cycle, duration from 24-42 days, patient has experienced bleeding since last ultrasound

---

Patient 9 “Odalis”

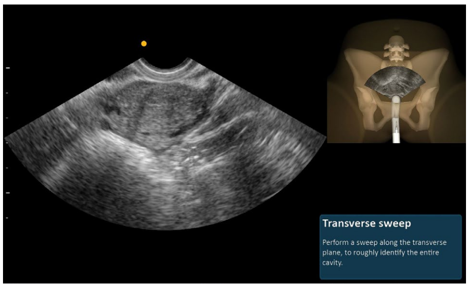

- Pregnancy classification: Pregnancy of unknown location
- Number of embryos: 0
- Actual gestational age: Unknown
- EGA based on LMP: Unknown
- Notes: Pregnancy of unknown location with a mass in the left adnexa indicating a possible ectopic pregnancy.
- Landmarks not possible to visualize: Gestational sac, embryo/fetus, yolk sac, and cardiac activity are not present.
- Previous pregnancies: 1 Gravida, 0 Para, 0 c-section, 0 ectopic
- Previous ultrasound for current pregnancy: None
- Recent medical history: Positive pregnancy test

Patient 10 “Kiana”

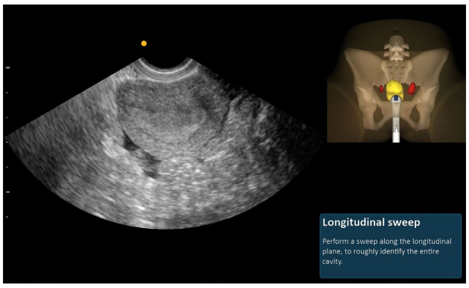

- Pregnancy classification: Pregnancy of unknown location
- Number of embryos: 0
- Actual gestational age: Unknown
- EGA based on LMP: Unknown
- Notes: Pregnancy of unknown location with a mass in the left adnexa indicating a possible ectopic pregnancy.
- Landmarks not possible to visualize: Gestational sac, embryo/fetus, yolk sac, and cardiac activity not present.
- Previous pregnancies: 1 Gravida, 0 Para, 0 c-section, 0 ectopic
- Previous ultrasound for current pregnancy: None
- Recent medical history: Positive pregnancy test

Patient 11 “Imani”

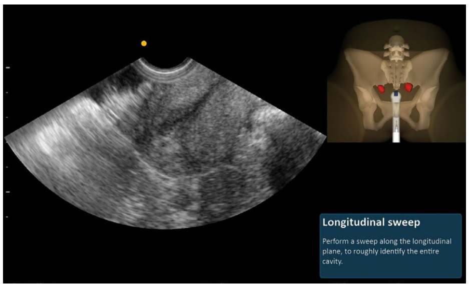

- Pregnancy classification: Pregnancy of unknown location
- Number of embryos: 0
- Actual gestational age: Unknown
- EGA based on LMP: Unknown
- Notes: Signs of ectopic pregnancy, fluid in the cul-de-sac, echolucent/sonolucent fluid or blood in the uterus
- Landmarks not possible to visualize: gestational sac, embryo/fetus, yolk sac, and cardiac activity are not present.
- Previous pregnancies: 1 Gravida, 0 Para, 0 c-section, 0 ectopic
- Previous ultrasound for current pregnancy: None
- Recent medical history: Positive pregnancy test

Patient 12 “Marina”

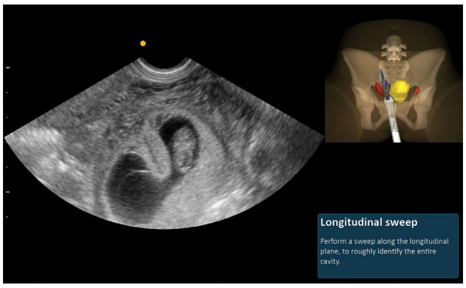

- Pregnancy classification: Ectopic
- Number of embryos: Multiple
- Actual gestational age: 8w 4d
- EGA based on LMP: 7w, 4d
- Notes: Visible ectopic twins. One embryo has a visible crown-rump length (CRL), which is measurable at 8w, 4d. The other embryo is not easily visualized. Gestational sac cannot be measured correctly.
- Landmarks not possible to visualize: All are possible to visualize.
- Previous pregnancies: 1 Gravida, 0 Para, 0 c-section, 0 ectopic
- Previous ultrasound for current pregnancy: None
- Recent medical history: Positive pregnancy test

Patient 13 “Vanessa”

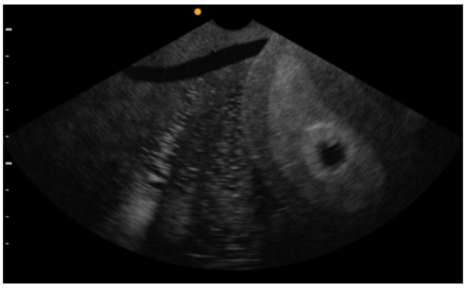

- Pregnancy classification: IUP
- Number of Embryos: 0
- Gestational age and/or related notes: 5week 3 days
- Landmarks not possible to visualize: none
- EGA based on LMP: 5 weeks
- Previous Pregnancies: 3
- Previous ultrasound for current pregnancy: no
- Recent Medical history: none

Patient 14 “Toni”

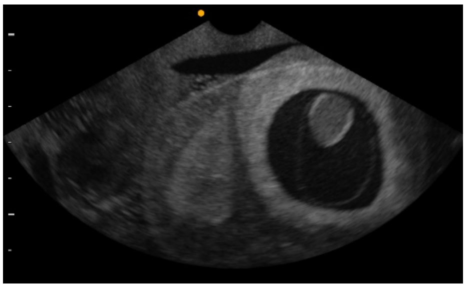

- Pregnancy classification: IUP
- Number of Embryos: 1
- Gestational age and/or related notes: 9 weeks 2 days
- Landmarks not possible to visualize: bicornuate is only seen in transverse plane
- EGA based on LMP: 9 weeks 6 days
- Previous Pregnancies: none
- Previous ultrasound for current pregnancy: no
- Recent Medical history: none

Patient 15 “Katy”

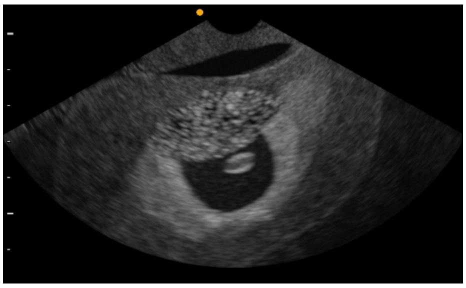

- Pregnancy classification: IUP/Possible Molar Pregnancy
- Number of Embryos: 1
- Gestational age and/or related notes: 7 weeks 3 days
- Landmarks not possible to visualize: clear endometrium (stripe), no cardiac
- EGA based on LMP: 8 weeks 5 days
- Previous Pregnancies: 3
- Previous ultrasound for current pregnancy: none
- Recent Medical history: severe N/V, and spotting

Patient 16 “Mei”

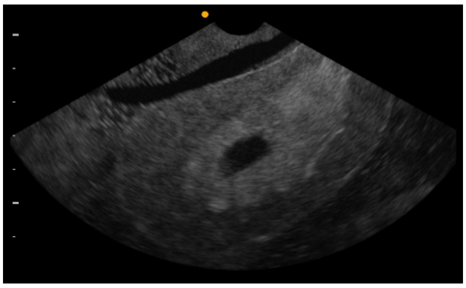

- Pregnancy classification: Possible ectopic
- Number of Embryos: 0
- Gestational age and/or related notes: 7 weeks 4 days
- Landmarks not possible to visualize: none
- EGA based on LMP: 7 weeks 4 days
- Previous Pregnancies: None
- Previous ultrasound for current pregnancy: none
- Recent Medical history: intermittent light bleeding and cramping x 2 weeks

Transvaginal obstetric ultrasound: testing mode

Patient 1 “Akira”

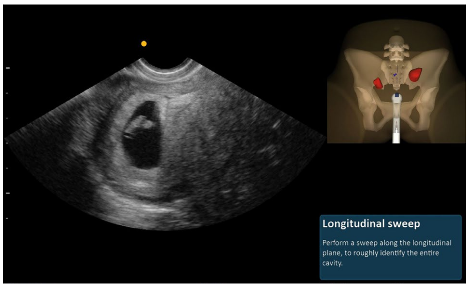

- Pregnancy classification: Definite intrauterine pregnancy
- Number of embryos: 1
- Actual gestational age: 7w, 1d
- EGA based on LMP: 6w, 3d
- Landmarks not possible to visualize: All are possible to visualize.
- Previous pregnancies: 3 Gravida, 2 Para, 0 c-section, 0 ectopic
- Previous ultrasound for current pregnancy: None
- Recent medical history: N/A

Patient 2 “Dakota”

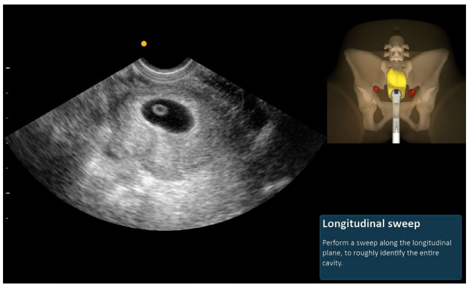

- Pregnancy classification: Early pregnancy loss (based on history only)
- Number of embryos: 0
- Actual gestational age: approx. 7w, 3d
- EGA based on LMP: 8w, 4d
- Notes: Spontaneous abortion, mean sac diameter measures at approx. 7w 3d, minimal grow since last ultrasound.
- Landmarks not possible to visualize: Embryo/fetus not present. No cardiac activity is present.
- Previous pregnancies: 2 Gravida, 1 Para, 0 c-section, 0 ectopic
- Previous ultrasound for current pregnancy: Yes, scanned 2 weeks ago at 6w, 6d
- Recent medical history: Irregular cycle, duration from 24-42 days, patient has experienced bleeding since last ultrasound

---

Patient 3 “Riley”

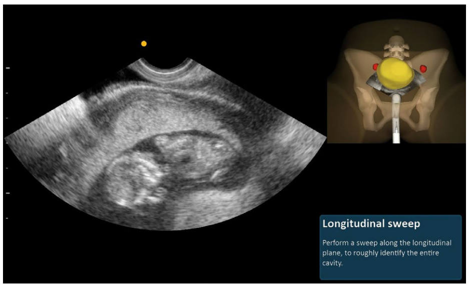

- Pregnancy classification: Definite intrauterine pregnancy
- Number of embryos: 1
- Actual gestational age: 12w, 1d
- EGA based on LMP: 11w, 5d
- Landmarks not possible to visualize: Yolk sac is not present.
- Previous pregnancies: 2 Gravida, 1 Para, 1 c-section, 0 ectopic
- Previous ultrasound for current pregnancy: None
- Recent medical history: N/A

Patient 4 “Imani”

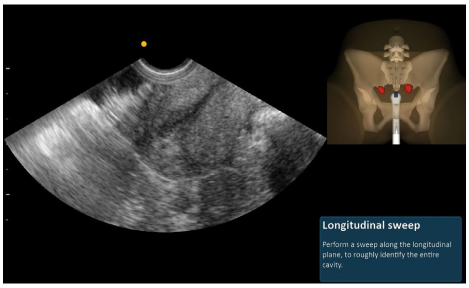

- Pregnancy classification: Pregnancy of unknown location
- Number of embryos: 0
- Actual gestational age: Unknown
- EGA based on LMP: Unknown
- Notes: Signs of ectopic pregnancy, fluid in the cul-de-sac, echolucent/sonolucent fluid or blood in the uterus
- Landmarks not possible to visualize: Gestational sac, embryo/fetus, yolk sac, and cardiac activity are not present
- Previous pregnancies: 1 Gravida, 0 Para, 0 c-section, 0 ectopic
- Previous ultrasound for current pregnancy: None
- Recent medical history: Positive pregnancy test

Patient 5 “Chante”

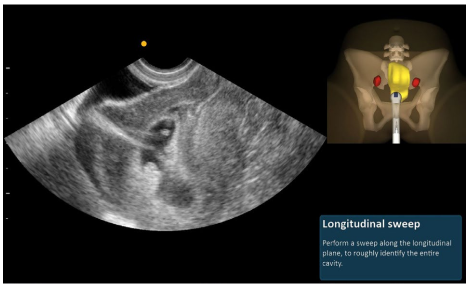

- Pregnancy classification: Early pregnancy loss
- Number of embryos: 1
- Actual gestational age: 6w, 6d
- EGA based on LMP: 6w, 6d
- Landmarks not possible to visualize: All are possible to visualize.
- Previous pregnancies: 1 Gravida, 0 Para, 0 c-section, 0 ectopic
- Previous ultrasound for current pregnancy: None
- Recent medical history: N/A

Patient 6 “Noel”

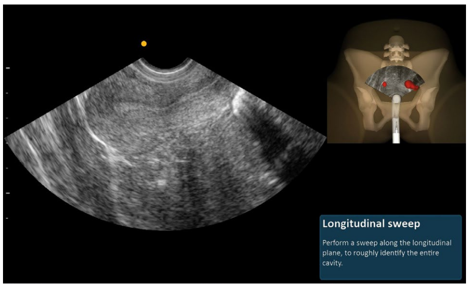

- Pregnancy classification: Pregnancy of unknown location
- Number of embryos: 0
- Actual gestational age: Unknown
- EGA based on LMP: 5w, 5d
- Notes: Pregnancy of unknown location. Patient could not be pregnant despite positive pregnancy test.
- Landmarks not possible to visualize: Gestational sac, embryo/fetus, yolk sac, and cardiac activity not present.
- Previous pregnancies: 1 Gravida, 0 Para, 0 c-section, 0 ectopic
- Previous ultrasound for current pregnancy: None
- Recent medical history: History of irregular menses, weekly positive pregnancy test

Patient 7 “Galia”

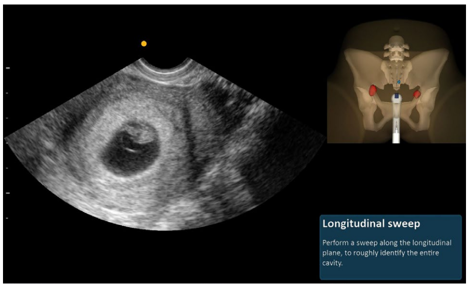

- Pregnancy classification: Definite intrauterine pregnancy
- Number of embryos: 1
- Actual gestational age: 8w, 1d
- EGA based on LMP: 8w, 1d
- Landmarks not possible to visualize: All are possible to visualize.
- Previous pregnancies: 1Gravida, 0 Para, 0 c-section, 0 ectopic
- Previous ultrasound for current pregnancy: None
- Recent medical history: N/A

Patient 8 “Kiana”

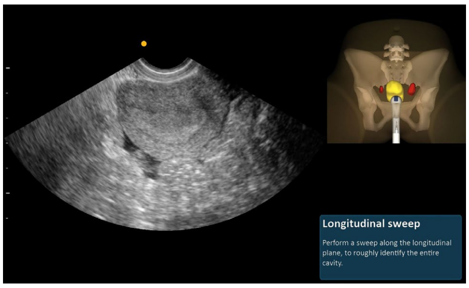

- Pregnancy classification: Pregnancy of unknown location
- Number of embryos: 0
- Actual gestational age: Unknown
- EGA based on LMP: Unknown
- Notes: Pregnancy of unknown location with a mass in the left adnexa indicating a possible ectopic pregnancy.
- Landmarks not possible to visualize: Gestational sac, embryo/fetus, yolk sac, and cardiac activity not present.
- Previous pregnancies: 1 Gravida, 0 Para, 0 c-section, 0 ectopic
- Previous ultrasound for current pregnancy: None
- Recent medical history: Positive pregnancy test

Patient 9 “Jasmine”

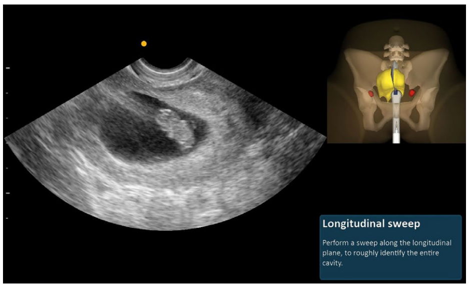

- Pregnancy classification: Definite intrauterine pregnancy
- Number of embryos: 1
- Actual gestational age: 9w, 1d
- EGA based on LMP: 9w, 1d
- Landmarks not possible to visualize: All are possible to visualize.
- Previous pregnancies: 1 Gravida, 0 Para, 0 c-section, 0 ectopic
- Previous ultrasound for current pregnancy: None
- Recent medical history: Nabothian cysts on cervix

Patient 10 “Sasha”

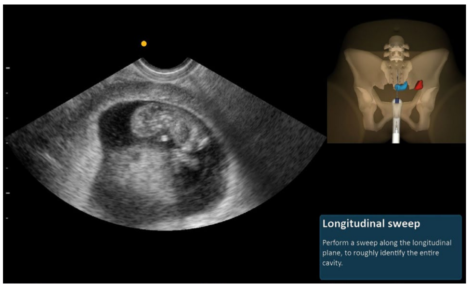

- Pregnancy classification: Definite intrauterine pregnancy
- Number of embryos: 1
- Actual gestational age: 10w
- EGA based on LMP: 13w, 2d
- Landmarks not possible to visualize: All are possible to visualize.
- Previous pregnancies: 3 Gravida, 1 Para, 1 c-section, 0 ectopic
- Previous ultrasound for current pregnancy: None
- Recent medical history: N/A

Patient 11 “Marina”

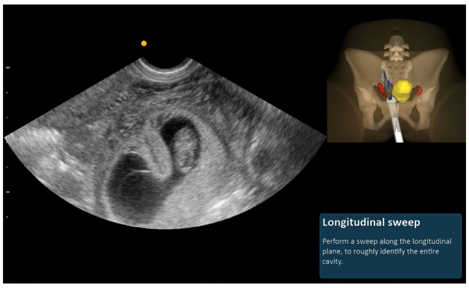

- Pregnancy classification: Ectopic
- Number of embryos: Multiple
- Actual gestational age: 8w 4d
- EGA based on LMP: 7w, 4d
- Notes: Visible ectopic twins. One embryo has a visible crown-rump length (CRL), which is measurable at 8w, 4d. The other embryo is not easily visualized. Gestational sac cannot be measured correctly.
- Landmarks not possible to visualize: All are possible to visualize.
- Previous pregnancies: 1 Gravida, 0 Para, 0 c-section, 0 ectopic

---

Patient 12 “Odalis”

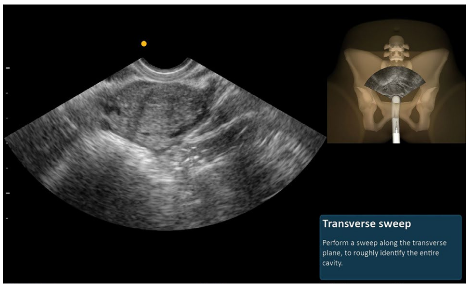

- Pregnancy classification: Pregnancy of unknown location
- Number of embryos: 0
- Actual gestational age: Unknown
- EGA based on LMP: Unknown
- Notes: Pregnancy of unknown location with a mass in the left adnexa indicating a possible ectopic pregnancy.
- Landmarks not possible to visualize: Gestational sac, embryo/fetus, yolk sac, and cardiac activity are not present.
- Previous pregnancies: 1 Gravida, 0 Para, 0 c-section, 0 ectopic
- Previous ultrasound for current pregnancy: None
- Recent medical history: Positive pregnancy test

Patient 13 “Mei”

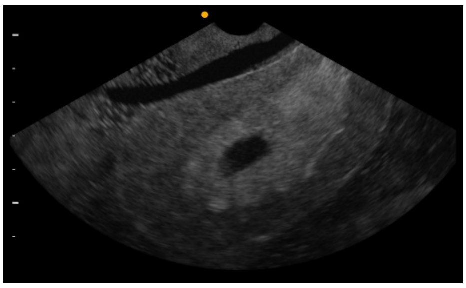

- Pregnancy classification: Possible ectopic
- Number of Embryos: 0
- Gestational age and/or related notes: 7 weeks 4 days
- Landmarks not possible to visualize: none
- EGA based on LMP: 7 weeks 4 days
- Previous Pregnancies: None
- Previous ultrasound for current pregnancy: none
- Recent Medical history: intermittent light bleeding and cramping x 2 weeks

Patient 14 “Toni”

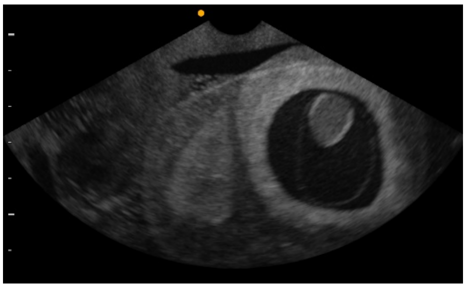

- Pregnancy classification: IUP
- Number of Embryos: 1
- Gestational age and/or related notes: 9 weeks 2 days
- Landmarks not possible to visualize: bicornuate is only seen in transverse plane
- EGA based on LMP: 9 weeks 6 days
- Previous Pregnancies: none
- Previous ultrasound for current pregnancy: no
- Recent Medical history: none

Patient 15 “Katy”

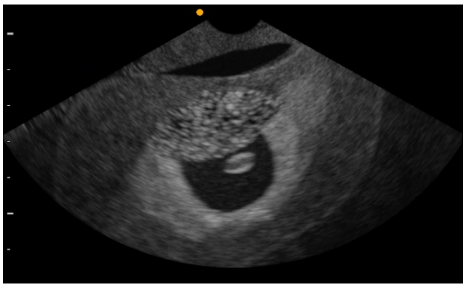

- Pregnancy classification: IUP/Possible Molar Pregnancy
- Number of Embryos: 1
- Gestational age and/or related notes: 7 weeks 3 days
- Landmarks not possible to visualize: clear endometrium (stripe), no cardiac
- EGA based on LMP: 8 weeks 5 days
- Previous Pregnancies: 3
- Previous ultrasound for current pregnancy: none
- Recent Medical history: severe N/V, and spotting

Patient 16 “Vanessa”

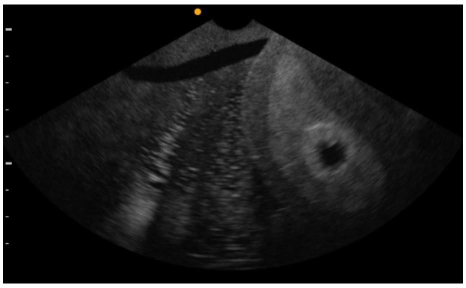

- Pregnancy classification: IUP
- Number of Embryos: 0
- Gestational age and/or related notes: 5week 3 days
- Landmarks not possible to visualize: none
- EGA based on LMP: 5 weeks
- Previous Pregnancies: 3
- Previous ultrasound for current pregnancy: no
- Recent Medical history: none
