# Supplementary material for: Development and evaluation of a “simulator-based” ultrasound training program for university teaching in obstetrics and gynecology–the prospective GynSim study
Source: Front Med (Lausanne). 2024 Apr 24;11:1371141. doi: 10.3389/fmed.2024.1371141 (PMC11076731; doi:10.3389/fmed.2024.1371141)
Supplement: Supplementary file 2 [file Data_Sheet_2.pdf]

Pregnancy

A pregnancy is divided into **three time periods**, called trimesters: the first (4 + 0 – 13 + 6 weeks of gestation), the second (14 + 0 – 25 + 6 weeks of gestation) and the third trimester (26 + 0 – 40 + 0 weeks of gestation).

High-frequency transvaginal ultrasound transducers should be preferred when assessing an early pregnancy. After 11 + 0 weeks of pregnancy however, the uterus is already large enough and a transabdominal approach leads to better visualization of the pregnancy . The zoom function allows you to enlarge the region of interest. Transvaginal sonography should be performed with an empty urinary bladder. On the contrary, a full bladder is preferrable when performing a transabdominal ultrasound scan, since it serves as an acoustic window, and improves the visualization of the uterus that lies behind it, especially if the latter is retroverted.

An ultrasound scan in early pregnancy should answer the following questions:

· Where is the pregnancy located?
· Is the pregnancy viable? (visualization of fetal cardiac activity)
· Number of embryos, chorionicity / amnionicity
· Determination of gestational age
· Detection of fetal abnormalities

Where is the pregnancy located?

An early ultrasound scan should confirm the intrauterine implantation of the pregnancy. The gestational sac is the first sonographic structure seen in an early intrauterine pregnancy. The gestational sac is an ovoid, asymmetrical, anechoic structure, surrounded by a hyperechoic rim and located eccentrically in the endometrium. The next sonographic sign of an early pregnancy is the yolk sac. The yolk sac is a circular, smooth walled structure with a maximum diameter of 6 mm (Fig. 1.1). The visualization of an intrauterine gestational sac with a yolk sac is considered a sign of a definitive intrauterine pregnancy.

The embryo is first visualized as a focal thickening at the edge of the yolk sac (Fig. 1.2). As the abdominal wall of the embryo closes during the process of granulation, the yolk sac is separated from it and is compressed between amnion and chorion. The embryo lies inside the amniotic cavity, while the yolk sac is always located outside the amnion (Fig. 1.3). The embryo is visible on ultrasound when it has a CRL of 1 – 2 mm. From this point onwards, it grows by approximately 1 mm per day. Cardiac activity should be visible in an embryo with a crown rump length (CRL) ≥ 7 mm. When measuring the CRL, the yolk sac should not be included in the measurement. A distinction between the cephalic and caudal end is only possible when the embryo is approximately 12 mm in size.

After 10 + 0 weeks of pregnancy, the embryo is called fetus. Organogenesis is complete at 13 + 0 weeks of gestation. Amnion and chorion fuse around 14 – 16 weeks of pregnancy.

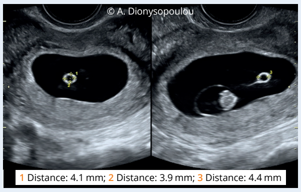

Fig.1.1 Yolc sac (transvaginal sonography)

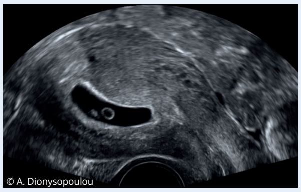

Fig.1.2 Yolc sac and embryo

Failed first-trimester pregnancy

The following criteria are helpful in order to distinguish between a viable and a nonviable early pregnancy:
· Gestational sac should be visualized from HCG levels of 1500 mIU/ml and more
· Yolc sac should be visualized from a gestational sac diameter of ≥ 10 mm
· Fetal cardiac activity should be visualized from a CRL ≥ 7 mm

Ultrasound criteria for a nonviable early pregnancy:
· Mean gestational sac diameter ≥ 25 mm without visible embryo
· Embryo with CRL ≥ 7 mm without cardiac activity (Fig. 1.5)
· Lack of embryo with cardiac activity 11 days after visualization of a gestational sac with yolk sac
· Lack of embryo with cardiac activity 14 days after visualization of a gestational sac without yolk sac
· Kein Wachstum einer Chrorionhöhle < 25 mm oder eines Embryos > 7 mm in 7 Tagen

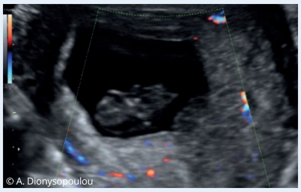

Fig.1.5 Pregnancy failure, 9 + 2. weeks of pregnancy (No visible cardiac motion)

Number of embryos

Once the diagnosis of an intrauterine pregnancy has been made, it is important to examine the entire pelvis in order to document the number of embryos. Müllerian gang anomalies can cause potential pitfalls. Multiple pregnancies, for example can implant in one or both horns of a bicornuate uterus. On the other hand, a subchorionic hematoma should not be mistaken for a second amniotic sac.

Twin pregnancies occur in approximately 1:85 of all pregnancies. Of these, 2 / 3 are dizygotic and genetically different, and 1 / 3 are monozygotic and therefore genetically identical. For the monitoring and management of multiple pregnancies, the correct determination of the chorionicity rather than the zygosity is crucial. **Dichorionic twins** are separated through a thick intertwin membrane. The chorionic tissue stretches between the separating amniotic membranes and forms the so-called λ (lamda) sign. In monochorionic twins, the separating membrane is very thin because it consists only of two layers of amnion. The attachment of the amnion to the common placenta is narrow and rectangular, forming a T-sign. If no separating membrane between the twins can be visualized, then this is a monochorionic monoamniotic pregnancy. Dichorionic twins are always diamniotic. This classification is a great determinant of outcome in multiple pregnancies. In cases of monochorionic twinning, parents should be informed about the risk of complications, like twin to twin transfusion syndrome (TTTS) and selective intrauterine growth restriction (sIUGR) due to vascular anastomoses related to monochorionicity and the pregnancy should be monitored accordingly.

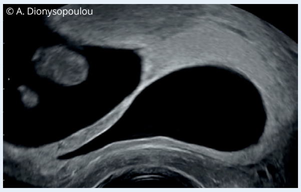

Fig.1.6 Dichorionic twin pregnancy, demonstration of the lamda sign

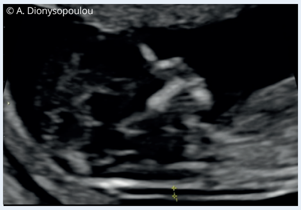

Fig.1.9 Nuchal translucency measurement

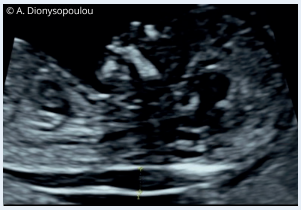

Fig.1.10 Increased nuchal translucency measurement

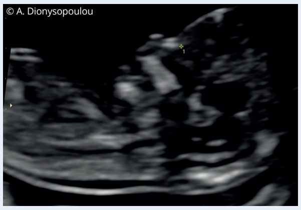

Fig.1.11 Nasal bone

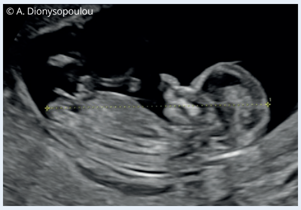

Fig.1.12 Measurement of the crown-rump length (CRL)

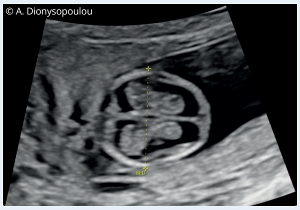

Fig.1.13 Measurement of the biparietal diameter

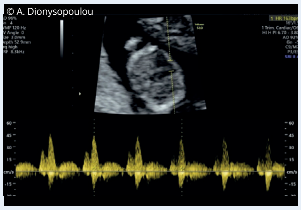

Fig.1.14 Measurement of the fetal heart rate

Ultrasound examination in the third trimester

The third trimester ultrasound scan serves to assess fetal wellbeing. For this purpose, the fetal growth, the amniotic fluid volume and the fetal and placental circulation are assessed. The fetal presentation and position of the placenta should also be documented.

Biometry

Different weight formulas can be used to estimate fetal weight. Hadlock´s formula is the one that is most commonly used. Four fetal biometric measurements are required for the calculation of the estimated fetal weight (EFW):
· Measurement of the biparietal diameter (BPD) (Fig.1.10)
· Measurement of the head circumference (HC) (Fig.1.10)
· Measurement of the abdominal circumference (AC) (Fig.1.11)
· Measurement of the femur length (FL) (Fig.1.12)

A discrepancy of up to 20% (in 5% of cases even over 20%) could lie between the estimated weight and the actual weight of the fetus. However, the aim fetal biometry is not to determine the absolute weight of the newborn, but rather to detect high-risk pregnancies affected by fetal growth restriction or fetal macrosomia, so that these can be monitored accordingly. The predictive accuracy of fetal biometry is believed to increase when serial ultrasound examinations are performed.

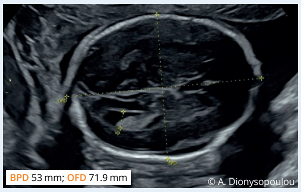

Fig.1.15 Biparietal (BPD) and occipitofrontal (OFD) diameter, Measurement of the lateral ventricle width (Vp)

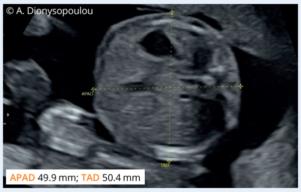

Fig.1.16 Transverse abdominal diameter- (TAD) and anterior- posterior abdominal diameter (APAD)

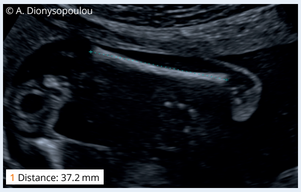

Fig.1.17 Measurement of the femur length

Fetal growth restriction (FGR)

Small for gestational age (SGA) is a fetus that is growing below the 5th or 10th percentile for this gestational age. The SGA fetus is not always a growth restricted fetus. The SGA fetus can be constitutionally small. This means that there is no underlying pathology; it is just a healthy, small fetus. An FGR fetus, on the other hand, is a fetus that has the genetical potential to be larger, but cannot grow adequately due to uteroplacental insufficiency. The FGR fetus is at risk of suffering intrauterine hypoxia. FGR fetuses are asymmetrically small due to particularly small abdominal circumference. Placental insufficiency leads

Workflow (Uterus und ovaries)

Purpose of the ultrasound scan

Exercise 4
Which **questions** should the sonographer answer when assessing the **uterus and ovaries**?

· Diagnostic modality of choice when examining the **uterus (often fibroids, cysts)**
· Obligatory diagnostic modality for the differential diagnosis of **pelvic pain**
· Detection of the **localization of the pregnancy (including extrauterine pregnancy) and monitoring of its progress**
· Detection of fluid accumulation in the pouch of **Douglas**

Which transducer type?

Exercise 5
Which **transducers** are used for the sonographic evaluation of the **uterus and ovaries** and why?

Convex transducer	Transvaginal transducer
Frequency of 3–5 MHz → offers the necessary penetration depth and serves as the first diagnostic orientation tool	transvaginal sonography is used for a more detailed examination of the female pelvic organs! This allows the organs to be displayed closer to the transducer. Higher frequencies (frequency range: 5–10 MHz) enable better image resolution!

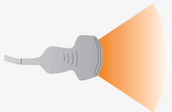

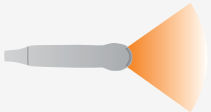

Sonographic reference planes

For the sonographic assessment of uterus and ovaries, suprapubic sagittal and transverse planes, as well as sagittal planes (parallel to the iliac vessels) are required! The organs should be visualized completely from their cranial to caudal and medial to lateral end.

Note
A **full bladder** is beneficial when performing a **transabdominal examination**! However, **transvaginal sonography** should be performed with an **empty urinary bladder**.

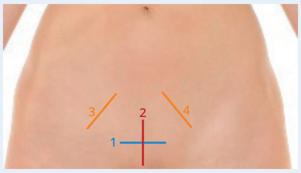

Fig.2.1 Scanning reference planes

1 Urinary Bladder and uterus, suprapubic transverse plane (cross-section); 2 Urinary bladder and uterus, suprapubic sagittal plane (longitudinal view); 3+4 Sagittal plane parallel to the iliac vessels

Uterine dimensions

Note
The craniocaudal **length (5–8 cm)** and the ventrodorsal **depth (1.5–3 cm)** of the uterus are measured in the sagittal view. The **width (3–5 cm)** can be determined in the transverse view. Even though transabdominal sonography can be used to perform measurements, transvaginal sonography provides better accuracy. The size of the uterus largely depends on whether the patient is parous or not.

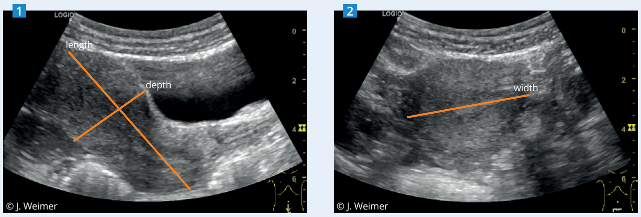

Fig.2.2 Uterine dimensions

Position of the uterus

The anatomical shape of the uterus resembles a pear turned upside down. The fundus uteri is the uppermost part of the uterine body (corpus uteri), above the entry points of the fallopian tubes in the sexually mature female. The uterine cervix is about one third of the total length of the non-gravid uterus. The description of the position of the uterus should always be included in the report. The most common form is the **combined anteversio and anteflexio**, in which the anterior wall of the uterus is tilted towards the urinary bladder. Furthermore, a right-to-left displacement from the median plane can occur, which can be described as dextroposition or levoposition of the uterus. Further position variants are: Anteversio + Retroflexio; Retroversio + Anteflexio and Retroversio + Retroflexio (see section Pathologies).

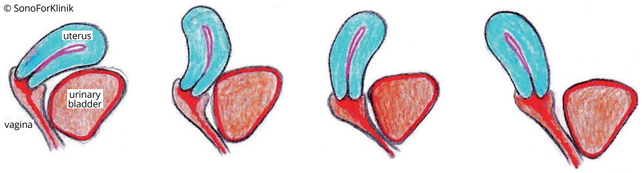

1 Anteversio + Anteflexio 2 Anteversio + Retroflexio 3 Retroversio + Anteflexio 4 Retroversio + Retroflexio

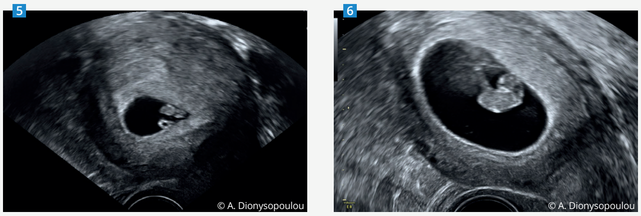

5 Early pregnancy 6 Early pregnancy

Ultrasound assessment of the ovaries

Note
The ovaries can be visualized transabdominally in a **cranio-lateral longitudinal section of the lower abdomen**. In **color doppler mode** you can often see the **direct proximity** to the **ovarian or iliac vessels**. The appearance of the ovaries depends on the phase of the menstrual cycle that the ultrasound scan is performed. The **size**, the **follicular status** (tertiary follicle or Graafian follicle present as a cyst-like anechoic structure; DD: ovarian cysts / polycystic ovary) should be determined, as well as possible **masses** (e.g. ovarian cancer, ovarian cysts) should be assessed. The detailed / routine ultrasound diagnosis is carried out using **transvaginal sonography**!

Exercise 10
Try to recognize the **ovaries** on the following ultrasound images **(sagittal plane)**. Notice the hypoechoic follicles and the proximity to the iliac vessels and the urinary bladder!

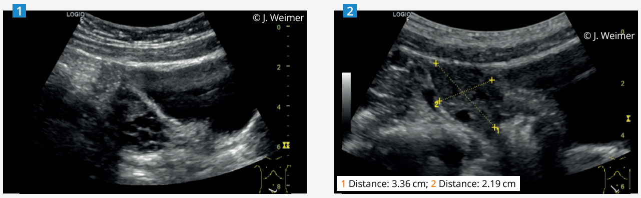

Exercise 11
Try to recognize the **different positions of the ovaries** on the following ultrasound images.

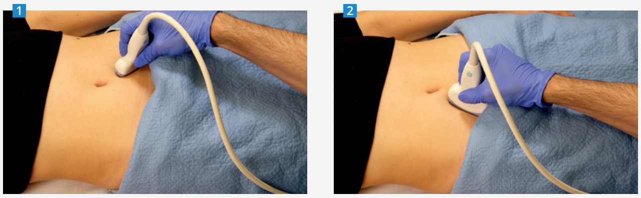

Exercise 12
Try to recognize the **ovaries** on the following ultrasound images **(transvaginal sonography)**. Pay attention to the hypoechoic follicles!

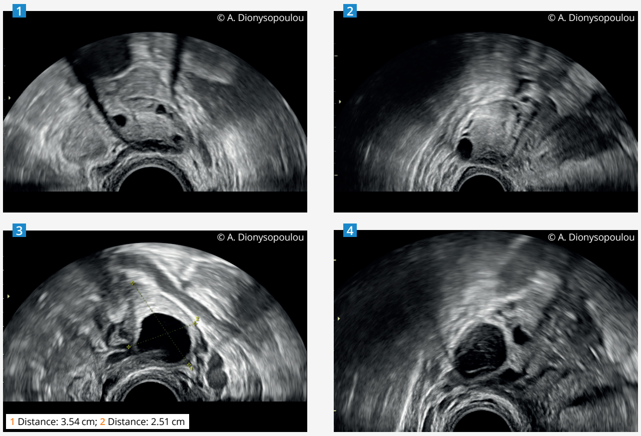

Pathological findings: Ovarian cysts

Functional cysts are the most common ovarian cysts. These are usually **follicular or corpus luteum cysts** and almost exclusively affect women in childbearing age. If a follicle does not release an egg and keeps growing, a follicular cyst is built. Corpus luteum cysts occur due to bleeding inside the rests of the Graafian follicle. In most cases, the functional cysts are a few centimeters in size and resolve spontaneously. However, any functional cyst can bleed **("hemorrhagic ovarian cyst")** or rupture spontaneously **("ruptured**
