# Supplementary material for: Development and evaluation of a “simulator-based” ultrasound training program for university teaching in obstetrics and gynecology–the prospective GynSim study
Source: Front Med (Lausanne). 2024 Apr 24;11:1371141. doi: 10.3389/fmed.2024.1371141 (PMC11076731; doi:10.3389/fmed.2024.1371141)
Supplement: Supplementary file 3 [file Data_Sheet_3.pdf]

Sample questions of the topic "Uterus"

The following ultrasound image shows:

- ☐ Uterus is transverse plane
- ☐ Uterus in sagittal plane
- ☐ Measurement of the endometrial thickness
- ☐ Solid ovarian mass
- ☐ All of the above are wrong.
- ☐ All of the above are correct.

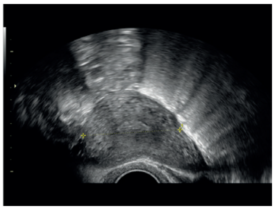

Sample questions of the topic "Douglas Pouch"

Which number represents the Douglas Pouch in the following ultrasound image:

- ☐ 1
- ☐ 2
- ☐ 3
- ☐ All of the above are wrong.

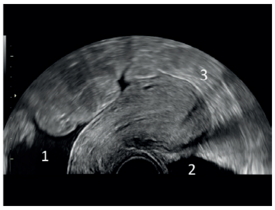

Sample questions of the topic "Ovaries and ovarian pathology"

The arrow on the following ultrasound image is showing:

- ☐ Pathologically enlarged ovary
- ☐ Ovary of a normal size
- ☐ Ectopic pregnancy
- ☐ Free fluid in the pouch of Douglas

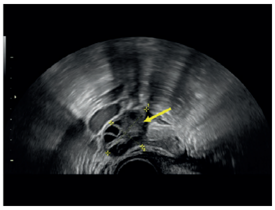

Sample questions of the topic "Ovaries and ovarian pathology"

A 22-year-old female patient presents in the emergency department with pelvic pain. The laboratory results are within the normal range. Pregnancy test is negative. You are performing a transvaginal ultrasound examination with the following finding. What is the most probable diagnosis?

- ☐ Haemorrhagic ovarian cyst
- ☐ Hydrosalpinx
- ☐ Tubo-ovarian abscess
- ☐ Uterine fibroid

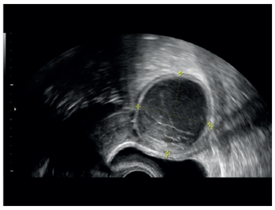

Sample questions of the topic "early pregnancy"

The following ultrasound image shows:

- ☐ Embryo with yolc sac
- ☐ Twin pregnancy
- ☐ Big ovarian cyst with internal echos
- ☐ All the above are wrong.

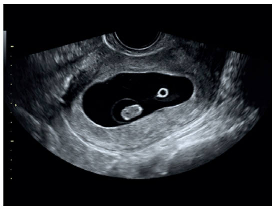

Sample questions of the topic "early pregnancy"

A 39-year-old patient presents with heavy vaginal bleeding in the 5+1 weeks of pregnancy. She has had two previous early pregnancy losses. The following ultrasound image shows:

- ☐ Intrauterine gestational sac
- ☐ Yolc sac
- ☐ Singleton pregnancy
- ☐ Twin pregnancy
- ☐ Big retrochorial hematoma
- ☐ Free fluid in the pouch of Douglas

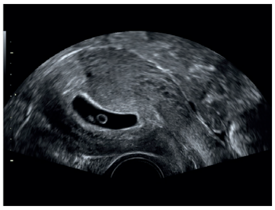

Sample questions of the topic "fetus and placenta"

Describe the position of the placenta:

- ☐ Anterior placenta
- ☐ Posterior placenta
- ☐ Lateral placenta
- ☐ The position of the placenta cannot be assessed in this ultrasound image.

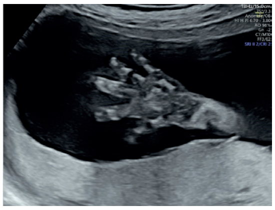

Sample questions of the topic "fetus and placenta"

A 33-year-old patient, presents in the 31 + 0 weeks of pregnancy with the suspicion of polyhydramnios. She has an insulin-dependent gestational diabetes. Her BMI is 38 kg/m². You perform an ultrasound examination and obtain the above sonographic findings. The estimated fetal weight corresponds to the 75th percentile. Which statement(s) is / are true?

- ☐ The amniotic fluid index (AFI) is 4,3 cm.
- ☐ A normal amniotic fluid index is 5 cm to 20 cm, thus this is an oligohydramnios.
- ☐ The measurement of the amniotic fluid depot was not performed correctly (no empty fluid pocket, fetal parts and umbilical cord are lying in the depot).
- ☐ This is a posterior placenta.
- ☐ In cases of gestational diabetes, where insulin does not lead to satisfactory glucose levels, an induction of u should be performed as soon as possible.

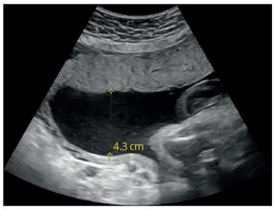

A

Sample questions of the topic "Uterus"

Please describe the position of the uterus in the picture above:

- ☐ Anteversio + Anteflexio
- ☐ Anteversio + Retroflexio
- ☐ Retroversio + Anteflexio
- ☐ Retroversio + Retroflexio

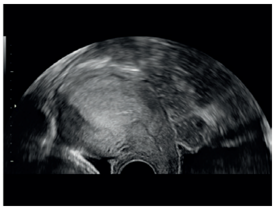

Sample questions of the topic "Douglas Pouch"

Which number represents the Douglas Pouch in the following ultrasound image:

- ☐ 1
- ☐ 2
- ☐ 3
- ☐ 4

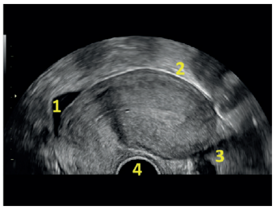

Sample questions of the topic "Ovaries and ovarian pathology"

The following ultrasound image is showing:

- ☐ Normal ovary with multiple follicles
- ☐ Multiple ovarian cysts
- ☐ Free fluid in the pouch of Douglas
- ☐ Corpus luteum cyst

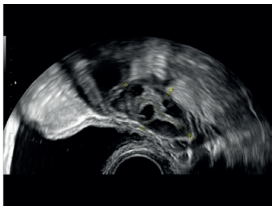

Sample questions of the topic "Ovaries and ovarian pathology"

The following ultrasound image shows a corpus luteum cyst. Which statement / -s is / are true?

- ☐ Corpus luteum cyst is the same as a teratoma
- ☐ Corpus luteum cysts are functional ovarian cysts
- ☐ Most corpus luteum cysts resolve spontaneously
- ☐ In the case of adnexal torsion, immediate surgical intervention is necessary

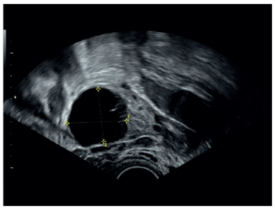

Sample questions of the topic "early pregnancy"

The following ultrasound images show an early pregnancy. Which image shows the correct measurement of the Crown rump length of the fetus?

- ☐ 1
- ☐ 2
- ☐ 3
- ☐ 4

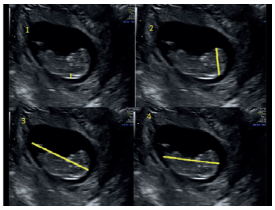

Sample questions of the topic "early pregnancy"

This is an ultrasound image of an early pregnancy. What does the arrow show?

Solution: ______________________

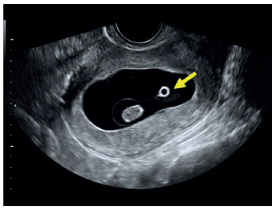

Sample questions of the topic "fetus and placenta"

Based on the following ultrasound image, please describe:

- The position of the placenta: ______________________
- The position of the umbilical cord insertion: ______________________

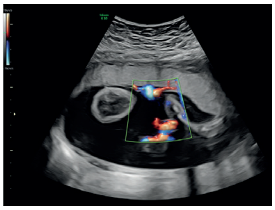

Sample questions of the topic "fetus and placenta"

A 29-year-old patient presents for an ultrasound examination in the 32 + 1 weeks of pregnancy. This is her first pregnancy. You perform a fetal biometry. The fetal abdominal circumference and estimated fetal weight is with 1450 g below the 3rd percentile. You estimate the amount of amniotic fluid performing the following measurement. Which statement / -s about the amount of amniotic fluid is / are true ?

- ☐ You used the Single deepest pockets (SDP) method.
- ☐ You used the Amniotic Fluid Index (AFI) method.
- ☐ Based on your measurement, the amount of amniotic fluid is within the normal range.
- ☐ Based on your measurement, this is an oligohydramnios

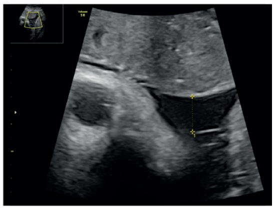

B
