# Supplementary figures and images for: Development and evaluation of a “simulator-based” ultrasound training program for university teaching in obstetrics and gynecology–the prospective GynSim study
Source: Front Med (Lausanne). 2024 Apr 24;11:1371141. doi: 10.3389/fmed.2024.1371141 (PMC11076731; doi:10.3389/fmed.2024.1371141)

A

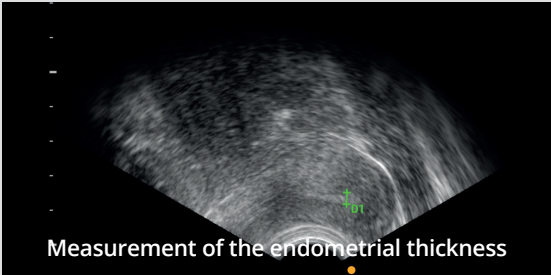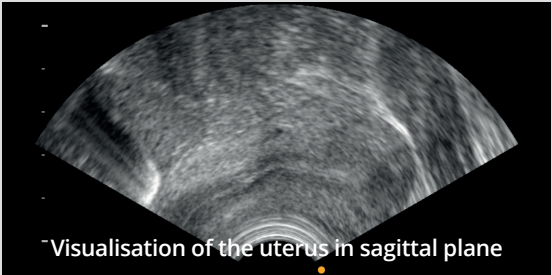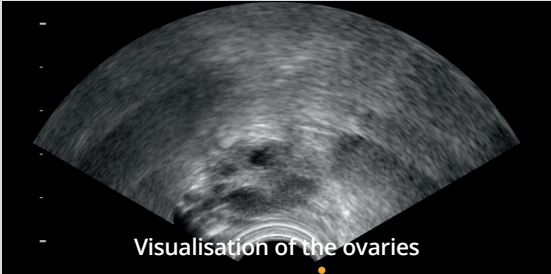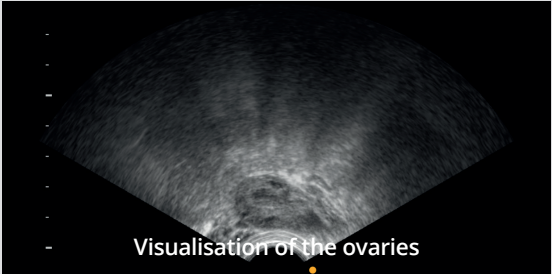

B

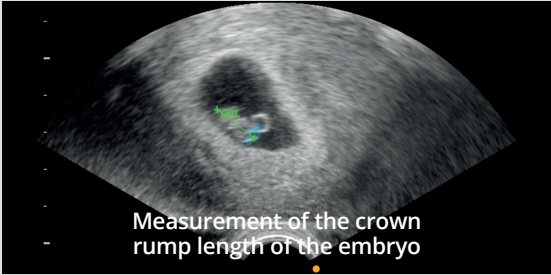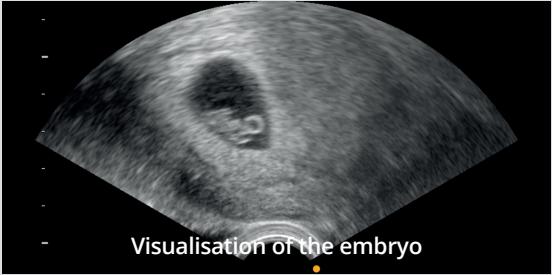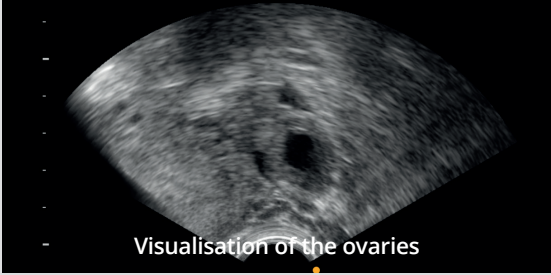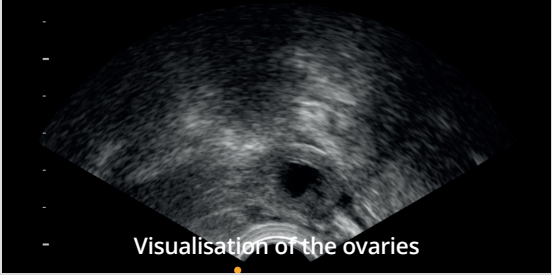

C

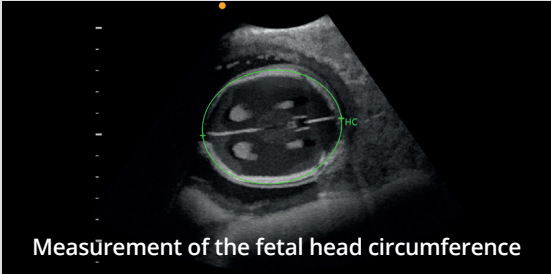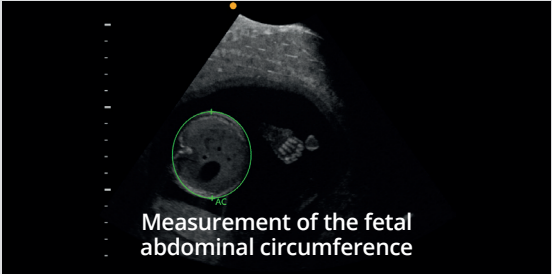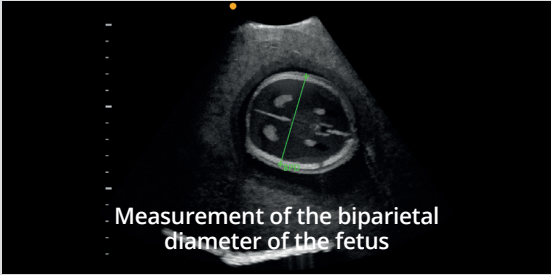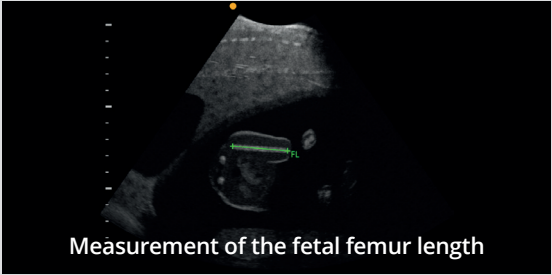

Supplement: Supplementary file 4 [file Data_Sheet_4.pdf]
