# Supplementary material for: Development and evaluation of a “simulator-based” ultrasound training program for university teaching in obstetrics and gynecology–the prospective GynSim study
Source: Front Med (Lausanne). 2024 Apr 24;11:1371141. doi: 10.3389/fmed.2024.1371141 (PMC11076731; doi:10.3389/fmed.2024.1371141)
Supplement: Supplementary file 5 [file Data_Sheet_5.PDF]

S5: Baseline characteristics of the participants; *n= number, ob/gyn: obstetric/ gynecologic, TAS: transabdominal ultrasound examinations, TVS transvaginal ultrasound examinations*

	**Control group T1 (n= 54)**	**Study group T1 (n= 59)**	***P – value***
Age; mean ± SD	27.5 ± 4.1	27.7 ± 3.3	*0.69*
Gender; n (%)<br>Male<br>Female	 23 (42.6%)<br>31 (57.4%)	 14 (23.7%)<br>45 (76.3%)	*0.05*
Previous apprenticeship; n (%)<br>Yes<br>No	 22 (40.7%)<br>32 (59.3%)	 39 (66.1%)<br>20 (33.9%)	*0.01*
Experience with ultrasound examinations in general; n (%)<br>Yes<br>No	 53 (100%)<br>0	 57 (96.6%)<br>2 (3.4%)	*0.52*
Number of ob/gyn TAS seen; mean ± SD	3.28 ± 2.23	3.24 ± 2.21	*0.92*
Number of ob/gyn TAS performed; mean ± SD	4.78 ± 2.11	4.03 ± 2.36	*0.08*
Number of ob/gyn TVS seen; mean ± SD	4.00 ± 2.22	3.56 ± 2.22	*0.29*
Number of ob/gyn TVS performed; mean ± SD	5.56 ± 1.41	4.86 ± 2.06	*0.04*
Previous contact with ultrasound simulators; n (%)<br>Yes<br>No	 1 (1.9%)<br>53 (98.1%)	 1 (1.7%)<br>58 (98.3%)	*1*
