# Supplementary material for: Development and evaluation of a “simulator-based” ultrasound training program for university teaching in obstetrics and gynecology–the prospective GynSim study
Source: Front Med (Lausanne). 2024 Apr 24;11:1371141. doi: 10.3389/fmed.2024.1371141 (PMC11076731; doi:10.3389/fmed.2024.1371141)
Supplement: Supplementary file 6 [file Data_Sheet_6.PDF]

S6: **Subjective competency assessment and level of self-confidence with regard to obstetric/gynecologic ultrasound examinations before and after finishing the Training program**; 7-point Likert scale (1= strongly disagree with the statement; 7= strongly agree with the statement)

T1: time point Evaluation^{pre}, upon enrollment, T2b: time point Evaluation^{post}, at the end of the course, T1-T2b: between time points T1 and T2b, SD: standard deviation, ob/gyn: obstetric/gynecologic, US: ultrasound, TAS: transabdominal ultrasound examinations, TVS transvaginal ultrasound examinations

	**Control group T1**	**Study group T1**	***P – value***	**Control group T2b**	**Study group T2b**	***P – value***	**Delta Control group**	**Delta Study group**	***Delta P-value***
							p-Value T1-T2b	*p-Value T1-T2b*	
**Subjective competecy assessment**									
	Mean ± SD	Mean ± SD		Mean ± SD	Mean ± SD		Mean ± SD	Mean ± SD	
							p-Value T1-T2b	*p-Value T1-T2b*	
Subjective competency in ob/gyn US	3.60 ± 0.96	3.33 ± 0.95	*0.14*	4.80 ± 1.01	5.11 ± 0.73	*0.11*	-1.22 ± 1.06	-1.7 ± 1.02	*0.03*
							(< 0.001)	*(< 0.001)*	
Knowledge of female anatomy	3.85 ± 1.14	3.64 ± 1.29	*0.37*	4.79 ± 1.09	5.20 ± 0.81	*0.04*	-1.00 ± 1.17	-1.59 ± 1.3	*0.02*
							(< 0.001)	*(< 0.001)*	
Spatial orientation in US imaging	3.70 ± 1.51	3.4 ± 1.12	*0.23*	4.86 ± 1.18	5.00 ± 1.03	*0.54*	-1.29 ± 1.6	-1.52 ± 1.3	*0.43*
							(< 0.001)	*(< 0.001)*	
Interpretation of US findings	3.65 ± 1.18	3.51 ± 1.32	*0.55*	4.86 ± 1.14	5.19 ± 0.8	*0.12*	-1.19 ± 1.38	-1.61 ± 1.41	*0.15*
							(< 0.001)	*(< 0.001)*	

Identification of anatomical structures on US	3.89 ± 1.3	3.68 ± 1.27	*0.38*	4.9 ± 1.17	5.42 ± 0.82	*0.02*	-1.05 ± 1.51<br>*(< 0.001)*	-1.68 ± 1.38<br>*(< 0.001)*	*0.04*
Identification of pathologic findings on US	2.83 ± 1.01	2.63 ± 1.26	*0,35*	4.62 ± 1.06	4.74 ± 1.01	*0.57*	-1.71 ± 1.1<br>*(< 0.001)*	-2.07 ± 1.3<br>*(< 0.001)*	*0.14*
**Level of self-confidence**									
Total score:<br>Level of self-confidence	1.78 ± 0.83	1.56 ± 0.85	0.17	5.14 ± 1.22	5.97 ± 1.13	*<0.001*	3.3 ± 1.4<br>*(< 0.001)*	-4.36 ± 1.47<br>*(< 0.001)*	*<0.001*
Comfort level in performing ob/gyn TVS independently	1.59 ± 0.79	1.44 ± 0.75	0.3	5.43 ± 1.38	6.06 ± 1.14	*0.02*	-3.74 ± 2.03<br>*(< 0.001)*	-4.57 ± 2.13<br>*(< 0.001)*	*0.01*
Comfort level in performing ob/gyn TAS independently	1.96 ± 1.1	1.68 ± 1.15	0.18	4.86 ± 1.24	5.89 ± 1.16	*<0.001*	-2.86 ± 1.74<br>*(< 0.001)*	-4.15 ± 1.39<br>*(< 0.001)*	*< 0.001*
