# Supplementary material for: Development and evaluation of a “simulator-based” ultrasound training program for university teaching in obstetrics and gynecology–the prospective GynSim study
Source: Front Med (Lausanne). 2024 Apr 24;11:1371141. doi: 10.3389/fmed.2024.1371141 (PMC11076731; doi:10.3389/fmed.2024.1371141)
Supplement: Supplementary file 7 [file Data_Sheet_7.PDF]

S7: Results of the students´ evaluations with regard to the following topic complexes: „Advantages of simulator-based ultrasound training“; „Motivation“ and „Learning goals“ between time points T1 (Evaluation^{pre}) and T2b (Evaluation^{post}); 7-point Likert scale (1= strongly disagree with the statement; 7= strongly agree with the statement)

T1: time point Evaluation^{pre}, upon enrollment, T2b: time point Evaluation^{post}, at the end of the course, T1-T2b: between time points T1 and T2b, SD: standard deviation, ob/gyn: obstetric/gynecologic, US: ultrasound

	**Control group T1**	**Study group T1**	***P – value***	**Control group T2b**	**Study group T2b**	***P – value***	**Delta Control group**	**Delta study group**	***Delta P - value***
							(p-Value T1-T2b)	*(p-Value T1-T2b)*	
**Advantages of simulator-based ultrasound training**									
	Mean ± SD	Mean ± SD		Mean ± SD	Mean ± SD		Mean ± SD	Mean ± SD	
							p-Value T1-T2	*p-Value T1-T2*	
Advantages of simulator-based ultrasound training, total score	5.98 ± 0.79	6.23 ± 0.78	*0.09*	5.7 ± 0.92	6.03 ± 0.81	*0.07*	0.35 ± 0.86	0.18 ± 1.01	*0.39*
							0.13	*0.18*	
Deepen theoretical knowledge	5.89 ± 1.31	6.32 ± 0.97	*0.05*	5.48 ± 1.41	6.17 ± 1.01	*0.01*	0.33 ± 1.41	0.19 ± 1.32	*0.6*
							0.15	*0.41*	
Teach practical skills	6.55 ± 0.82	6.66 ± 0.73	*0.44*	6.38 ± 1.08	6.72 ± 0.66	*0.08*	0.15 ± 0.96	-0.07 ± 0.87	*0.25*
							0.41	*0.64*	

Practice how to handle real patients	4.57 ±1.66	5.03 ± 1.87	*0.17*	3.86 ± 1.92	4.17 ± 1.92	*0.44*	0.95 ± 2.04<br>*0.06*	0.81 ± 2.14<br>*0.02*	*0.75*
Increase patient Safety	6.0 ± 1.04	6.22 ± 1.22	*0.3*	5.59 ± 1.5	6.02 ± 1.33	*0.15*	0.53 ± 1.47<br>*0.14*	0.15 ± 1.61<br>*0.4*	*0.24*
Increase comfort level with regard to performing ultrasound examinations independently	6.59 ± 0.77	6.68 ± 0.71	*0.54*	6.38 ± 1.06	6.5 ± 1.08	*0.59*	0.17 ± 1.01<br>*0.28*	0.15 ± 1.3<br>*0.31*	*0.94*
Increase motivation to learn	6.31 ± 0.91	6.47 ± 0.82	*0.33*	6.21 ± 0.93	6.59 ± 0.74	*0.03*	0.14 ± 0.87<br>*0.6*	-0.15 ± 1.07<br>*0.42*	*0.15*
**Motivation**									
Interest for ob/gyn simulator ultrasound training	5.48 ± 1.34	6.03 ± 1.04	***0.02***	5.75 ± 1.59	6.35 ± 1.02	*0.07*	-0.41 ± 1.78<br>*0.43*	-0.35 ± 1.18<br>*0.13*	*0.87*
**Learning goals**									
Learning goals	6.21 ± 0.82	6.62 ± 0.54	*0.002*	6.02 ± 0.82	6.21 ± 0.83	*0.27*	0.2 ± 0.79<br>*0.27*	0.41 ± 0.88<br>*0.002*	*0.24*
How to use US simulators	6.06 ± 1.45	6.53 ± 0.95	*0.05*	6.32 ± 0.96	6.48 ± 0.93	*0.4*	-0.22 ± 1.29<br>*0.29*	0.09 ± 1.23<br>*0.8*	*0.24*
	6.26 ± 1.25	6.69 ± 0.62		6.24 ± 0.86	6.46 ± 0.84		0.02 ± 1.3	0.2 ± 0.92	*0.4*

How to perform ob/gyn US examinations			*0.02*			*0.22*	*0.94*	*0.1*	
Knowledge of female anatomy	6.11 ± 1.18	6.51 ± 0.8	*0.04*	5.76 ± 1.04	5.96 ± 1.17	*0.37*	0.41 ± 1.05	0.57 ± 1.28	*0.51*
							0.12	*0.004*	
Identification of anatomical structures on US	6.28 ± 1	6.59 ± 1	*0.1*	5.95 ± 0.95	6.19 ± 0.97	*0.24*	0.29 ± 1.03	0.41 ± 1.06	*0.6*
							0.11	*0.03*	
Identification of pathologic findings on US	6.35 ± 0.83	6.69 ± 0.71	*0.02*	5.85 ± 1.01	5.98 ± 1.14	*0.57*	0.54 ± 1.07	0.68 ± 1.17	*0.54*
							0.01	< *0.001*	
