# Supplementary material for: Development and evaluation of a “simulator-based” ultrasound training program for university teaching in obstetrics and gynecology–the prospective GynSim study
Source: Front Med (Lausanne). 2024 Apr 24;11:1371141. doi: 10.3389/fmed.2024.1371141 (PMC11076731; doi:10.3389/fmed.2024.1371141)
Supplement: Supplementary file 8 [file Data_Sheet_8.PDF]

S8: Results of the students´evaluations with regard to the „Teaching materials“, the „Properties of the ultrasound Simulator“ and “Future perspectives and attitudes towards simulator-based ultrasound training in Obstetrics and Gynecology“ at time point T2b (Evaluation^{post}); 7-point Likert scale (1= strongly disagree with the statement; 7= strongly agree with the statement)

SD: standard deviation, ob/gyn: Obstetrics and Gynecology

	**Control group (Mean ± SD)**	**Study group (Mean ± SD)**	***P – value***
**Teaching materials**			
Studied the lecture notes	3.23 ± 1.84	3.98 ± 2	*0.06*
Satisfied with the lecture notes	5.38 ± 1.29	5.54 ± 1.13	*0.55*
**Properties of the ultrasound simulator**			
Properties of the ultrasound simulator, total score	6.2 ± 0.87	6.29 ± 0.81	*0.64*
Handling	6.07 ± 1.22	6.02 ± 1.02	*0.82*
Haptics	6.29 ± 0.97	6.3 ± 1.18	*0.96*
Image quality	6.1 ± 0.96	6.33 ± 0.91	*0.22*
Presentation and realistic visualization of anatomical structures	6.31 ± 0.98	6.43 ± 0.82	*0.54*
Presentation and realistic visualization of pathologic findings	6.26 ± 0.91	6.35 ± 0.94	*0.64*
**Future perspectives and attitudes towards simulator-based ultrasound training in ob/gyn**			
Comparison Training on the ultrasound simulator vs Training on real patients	1.4 ± 0.59	1.56 ± 0.98	*0.35*
Increase one´s understanding with regard to pathologic findings on ultrasound	5.57 ± 1.35	6.19 ± 0.99	*0.02*
Increase one´s interest in the field of Obstetrics and Gynecology	4.29 ± 1.69	5.11 ± 1.54	*0.02*
The Training on ultrasound simulators is a useful aiding tool to medical training	6.38 ± 1.16	6.93 ± 0.33	*0.01*
Simulator-based ultrasound training should be integrated in the ob/gyn curriculum	6.47 ± 1.02	6.89 ± 0.38	*0.03*
