# Supplementary material for: Development and evaluation of a “simulator-based” ultrasound training program for university teaching in obstetrics and gynecology–the prospective GynSim study
Source: Front Med (Lausanne). 2024 Apr 24;11:1371141. doi: 10.3389/fmed.2024.1371141 (PMC11076731; doi:10.3389/fmed.2024.1371141)
Supplement: Supplementary file 9 [file Data_Sheet_9.PDF]

S9: Results of the practical test on the ultrasound simulator at time point T2a; N= number of measurements that could be taken into account in the analysis

Faktor	Control group	Study group	*p-value*
	Mean ± SD	Mean ± SD	
	[N (%)]	[N (%)]	
Total score_Practical test	0.78 ± 0.14	0.92 ± 0.12	< *0.001*
Total score module 1	0.78 ± 0.17	0.93 ± 0.1	< *0.001*
	[53 (48.18%)]	[57 (51.81%)]	
Total score module 2	0.92 ± 0.13	0.97 ± 0.1	*0.02*
	[54 (48.65%)]	[57 (51.35%)]	
Total score module 3	0.86 ± 0.19	0.95 ± 0.12	*0.001*
	[54 (47.79%)]	[59 (52.21%)]	
Qualitative assessment: Identify and demonstrate the required anatomical level and structures	0.85 ± 0.1	0.95 ± 0.07	< *0.001*
	[53 (48.18%)]	[57 (51.81%)]	
Total score: correct interpretation of findings	0.72 ± 0.25	0.88 ± 0.22	< *0.001*
	[54 (48.21%)]	[58 (51.79%)]	
Module 1: Interpretation of findings (wrong: 0, correct: 1)			*0.001*
Module 2:Interpretation of findings (wrong:0,correct:1)			*0.71*
Total score deviation from the reference value	430.73 ± 15.62	436.51 ± 16.65	*0.24*
	[18 (40%)]	[27 (60%)]	
Deviation from the reference value (absolute value)	18.83 ± 12.90	14.65 ± 13.4	*0.3*
	[18 (40%)]	[27 (60%)]	
Module 1: Measurement of the endometrial thickness in mm 3.3mm	3.42 ± 2.86	2.7 ± 0.99	*0.13*
	[42 (48.84%)]	[44 (51.16%)]	
Deviation	1.29 ± 2.55	0.95 ± 0.64	*0.41*

Module 2: Measurement of the CRL in mm (14.1 mm)	12.33 ± 2.37	12.58 ± 1.81	*0.56*
	[49 (47.57%)]	[54 (52.43%)]	
Deviation	2 ± 2.18	1.82 ± 1.51	*0.64*
Module 3: Measurement of the BPD in mm (53.9 mm)	54.15 ± 3.34	51.83 ± 7.16	*0.05*
	[40 (44.44%)]	[50 (55.56%)]	
Deviation	1.29 ± 2.55	0.95 ± 0.64	*0.58*
Module 3: Measurement of the HC in mm (190.4 mm)	179.71 ± 10.24	181.62 ± 9.84	*0.35*
	[45 (45.45%)]	[54 (54.55%)]	
Deviation	11.84 ± 8.85	10.93 ± 7.33	*0.58*
Module 3: Measurement of the AC in mm (152.4 mm)	151.36 ± 20.01	154.84 ± 7.65	*0.29*
	[42 (45.65%)]	[50 (54.35%)]	
Deviation	8.48 ± 18.11	6.27 ± 4.95	*0.45*
Module 3: Measurement of the FL in mm (33.4 mm)	31.74 ± 3.08	35.37 ± 20.5	*0.19*
	[48 (45.71%)]	[57 (54.29%)]	
Deviation	2.11 ± 2.78	4.01 ± 2.02	*0.49*
Total deviation	419.06 ± 14.72	421.59 ± 16.59	*0.51*
	[27 (39.71%)]	[41 (60.29%)]	
Total score_Time to complete	1550.54 ±295.46	1480.91 ±300.56	*0.22*
	[52 (47.27%)]	[58 (52.73%)]	
Module 1: Time to complete (min:sec)	443.22 ± 117	405.37 ± 106.7	*0.08*
	[54 (47.79%)]	[59 (52.21%)]	
Module 2: Time to complete (min:sec)	409.61 ± 113.28	372.63 ± 113.38	*0.09*
	[54 (47.79%)]	[59 (52.21%)]	
Module 3: Time to complete (min:sec)	701.04 ± 158.7	710.97 ± 169.6	*0.75*
	[52 (47.27%)]	[58 (52.73%)]	
