# Supplementary material for: Development and evaluation of a “simulator-based” ultrasound training program for university teaching in obstetrics and gynecology–the prospective GynSim study
Source: Front Med (Lausanne). 2024 Apr 24;11:1371141. doi: 10.3389/fmed.2024.1371141 (PMC11076731; doi:10.3389/fmed.2024.1371141)
Supplement: Supplementary file 10 [file Data_Sheet_10.PDF]

S10: Listing of the analyzed possible influencing factors on the results of the theoretical and practical tests

Test Part	Potential influencing factors
**Theory ^{Pre}**	
	Age
	gender: (male, female)
	onsite training course on the ultrasound simulator (yes/no)
	number of obstetric/gynecologic transabdominal ultrasound examinations seen
	number of obstetric/gynecologic transabdominal ultrasound examinations performed
	number of obstetric/gynecologic transvaginal ultrasound examinations seen
	number of obstetric/gynecologic transvaginal ultrasound examinations performed
	subjective competency in obstetric/gynecologic ultrasound at time point T1
	interest for obstetric/gynecologic simulator ultrasound training at time point T1
**Theory^{post}**	
	total score on the Test Theory^{pre}
	subjective competency in obstetric/gynecologic ultrasound at time point T2b
	studied the lecture notes
	interest for obstetric/gynecologic simulator ultrasound training at time point T2b
	total score on the Practical Test^{post}
**Practical Test^{post}**	
	age
	gender: (male, female)
	onsite training course on the ultrasound simulator (yes/no)
	number of obstetric/gynecologic transabdominal ultrasound examinations seen
	number of obstetric/gynecologic transabdominal ultrasound examinations performed
	number of obstetric/gynecologic transvaginal ultrasound examinations seen
	number of obstetric/gynecologic transvaginal ultrasound examinations performed
	subjective competency in obstetric/gynecologic ultrasound at time point T1
	subjective competency in obstetric/gynecologic ultrasound at time point T2b
	interest for obstetric/gynecologic simulator ultrasound training at time point T1
	total score on the Test Theory^{pre}
	total score on the Test Theory^{post}
	studied the lecture notes
	interest for obstetric/gynecologic simulator ultrasound training at time point T2b
	increase one´s understanding with regard to pathologic findings on ultrasound
