# Supplementary material for: Development and evaluation of a “simulator-based” ultrasound training program for university teaching in obstetrics and gynecology–the prospective GynSim study
Source: Front Med (Lausanne). 2024 Apr 24;11:1371141. doi: 10.3389/fmed.2024.1371141 (PMC11076731; doi:10.3389/fmed.2024.1371141)
Supplement: Supplementary file 11 [file Data_Sheet_11.pdf]

S 11: Significant correlations of subjective and objective result; R= correlation coefficient, ob/gyn: obstetric/ gynecologic, TAS: transabdominal ultrasound examinations, TVS transvaginal ultrasound examinations, T1: Evaluation^{pre}, T2b: Evaluation^{post}

	Factor 1 (y)	Factor 2 (x)	R	p-value
subjective – subjective	Comfort level in performing ob/gyn TAS independently (T1)	Comfort level in performing ob/gyn TVS independently (T1)	0.55	< 0.001
subjective – subjective	Subjective level of skills competence in ob/gyn ultrasound(T1)	Comfort level in performing ob/gyn TAS independently (T1)	0.39	< 0.001
subjective – subjective	Comfort level in performing ob/gyn TAS independently (T1)	Subjective level of skills competence in ob/gyn ultrasound (T1)	0.68	< 0.001
subjective – subjective	Comfort level in performing ob/gyn TVS independently (T1)	Subjective level of skills competence in ob/gyn ultrasound (T1)	0.46	< 0.001
subjective – subjective	Subjective level of skills competence in ob/gyn ultrasound (T2b)	Subjective level of skills competence in ob/gyn ultrasound (T1)	0.32	0.001
subjective – subjective	Subjective level of skills competence in ob/gyn ultrasound (Delta)	Subjective level of skills competence in ob/gyn ultrasound (T1)	0.64	< 0.001
subjective – subjective	Subjective level of skills competence in ob/gyn ultrasound (Delta)	Comfort level in performing ob/gyn TAS independently (T1)	0.41	< 0.001
subjective – subjective	Learning golas (T1)	Interest for ob/gyn simulator ultrasound training (T1)	0.38	< 0.001
subjective – subjective	Advantages of simulator-based ultrasound Training-Total score (T1)	Interest for ob/gyn simulator ultrasound training (T1)	0.42	< 0.001
subjective – subjective	Subjective level of skills competence in ob/gyn ultrasound (T2b)	Interest for ob/gyn simulator ultrasound training (T1)	0.31	0.001
subjective – subjective	Interest for ob/gyn simulator ultrasound training (T2b)	Interest for ob/gyn simulator ultrasound training (T1)	0.52	< 0.001
subjective – subjective	Advantages of simulator-based ultrasound Training-Total score (T1)	Learning golas (T1)	0.45	< 0.001
subjective –	Learning golas (T2b)	Learning golas (T1)	0.41	< 0.001

subjective				
subjective – subjective	Subjective level of skills competence in ob/gyn ultrasound (T2b)	Advantages of simulator-based ultrasound Training-Total score (T1)	0.31	0.001
subjective – subjective	Learning golas (T2b)	Advantages of simulator-based ultrasound Training-Total score (T1)	0.33	0.001
subjective – subjective	Advantages of simulator-based ultrasound Training-Total score (Delta)	Advantages of simulator-based ultrasound Training-Total score (T1)	0.52	< 0.001
objective – objective	Test Theory^{post}-Total score (T2b)	Test Theory^{pre}-Total score (T1)	0.24	0.03
objective – objective	Total score Deviation	Test Theory^{pre}-Total score (T1)	-0.4	0.01
objective – objective	Theory Test-Total score (Delta)	Test Theory^{pre}-Total score (T1)	0.57	< 0.001
subjective – subjective	Comfort level in performing ob/gyn TAS independently (T2b)	Subjective level of skills competence in ob/gyn ultrasound (T2b)	0.54	< 0.001
subjective – subjective	Comfort level in performing ob/gyn TVS independently (T2b)	Subjective level of skills competence in ob/gyn ultrasound (T2b)	0.64	< 0.001
subjective – subjective	Interest for ob/gyn simulator ultrasound training (T2b)	Subjective level of skills competence in ob/gyn ultrasound (T2b)	0.44	< 0.001
subjective – subjective	Learning goals (T2b)	Subjective level of skills competence in ob/gyn ultrasound (T2b)	0.45	< 0.001
subjective – subjective	Advantages of simulator-based ultrasound Training-Total score (T2b)	Subjective level of skills competence in ob/gyn ultrasound (T2b)	0.47	< 0.001
subjective – subjective	Properties of the ultrasound simulator-Total score (T2b)	Subjective level of skills competence in ob/gyn ultrasound (T2b)	0.45	< 0.001
subjective – subjective	Improvement in visuomotor skills (T2b)	Subjective level of skills competence in ob/gyn ultrasound (T2b)	0.49	< 0.001
subjective – subjective	Comfort level in performing ob/gyn TVS inde-pendently (Delta)	Subjective level of skills competence in ob/gyn ultrasound (T2b)	0.34	< 0.001
subjective –	Comfort level in performing ob/gyn TVS inde-pendently	Comfort level in performing ob/gyn TAS	0.81	< 0.001

subjective	(T2b)	independently (T2b)		
subjective – subjective	Interest for ob/gyn simulator ultrasound training (T2b)	Comfort level in performing ob/gyn TAS independently (T2b)	0.38	0.003
subjective – subjective	Learning goals (T2b)	Comfort level in performing ob/gyn TAS independently (T2b)	0.57	< 0.001
subjective – subjective	Advantages of simulator-based ultrasound Training-Total score (T2b)	Comfort level in performing ob/gyn TAS independently (T2b)	0.54	< 0.001
subjective – subjective	Properties of the ultrasound simulator-Total score (T2b)	Comfort level in performing ob/gyn TAS independently (T2b)	0.47	< 0.001
subjective – subjective	Improvement in visuomotor skills (T2b)	Comfort level in performing ob/gyn TAS independently (T2b)	0.58	< 0.001
objektiv – subjektiv	Qualitative assessment Practical Test-Total score	Comfort level in performing ob/gyn TAS independently (T2b)	0.30	0.01
subjective – subjective	Comfort level in performing ob/gyn TAS independently (Delta)	Comfort level in performing ob/gyn TAS independently (T2b)	0.78	< 0.001
subjective – subjective	Comfort level in performing ob/gyn TVS independently (Delta)	Comfort level in performing ob/gyn TAS independently (T2b)	0.76	< 0.001
subjective – subjective	Interest for ob/gyn simulator ultrasound training (T2b)	Comfort level in performing ob/gyn TVS independently (T2b)	0.45	< 0.001
subjective – subjective	Learning goals (T2b)	Comfort level in performing ob/gyn TVS independently (T2b)	0.56	< 0.001
subjective – subjective	Advantages of simulator-based ultrasound Training-Total score (T2b)	Comfort level in performing ob/gyn TVS independently (T2b)	0.48	< 0.001
subjective – subjective	Properties of the ultrasound simulator-Total score (T2b)	Comfort level in performing ob/gyn TVS independently (T2b)	0.54	< 0.001
subjective – subjective	Improvement in visuomotor skills (T2b)	Comfort level in performing ob/gyn TVS independently (T2b)	0.6	< 0.001
subjective – subjective	Comfort level in performing ob/gyn TAS independently (Delta)	Comfort level in performing ob/gyn TVS independently (T2b)	0.51	< 0.001
subjective –	Comfort level in performing ob/gyn TVS independently	Comfort level in performing ob/gyn TVS	0.76	< 0.001

subjective	(Delta)	independently (T2b)		
subjective – subjective	Learning goals (T2b)	Interest for ob/gyn simulator ultrasound training (T2b)	0.41	< 0.001
subjective – subjective	Improvement in visuomotor skills (T2b)	Interest for ob/gyn simulator ultrasound training (T2b)	0.32	0.003
objective – subjective	Theory Test-Total score (T2b)	Interest for ob/gyn simulator ultrasound training (T2b)	0.32	0.002
objective – subjective	Qualitative assessment Practical Test-Total score	Interest for ob/gyn simulator ultrasound training (T2b)	0.30	0.01
subjective – subjective	Advantages of simulator-based ultrasound training (post)	Learning goals (T2b)	0.66	< 0.001
subjective – subjective	Properties of the ultrasound simulator-Total score (T2b)	Learning goals (T2b)	0.51	< 0.001
subjective – subjective	Improvement in visuomotor skills (T2b)	Learning goals (T2b)	0.48	< 0.001
subjective – subjective	Comfort level in performing ob/gyn TVS inde-pendently (Delta)	Learning goals (T2b)	0.5	< 0.001
subjective – subjective	Properties of the ultrasound simulator-Total score (T2b)	Advantages of simulator-based ultrasound Training-Total score (T2b)	0.51	< 0.001
subjektiv – subjektiv	Improvement in visuomotor skills (T2b)	Advantages of simulator-based ultrasound Training-Total score (T2b)	0.41	< 0.001
subjective – subjective	Improvement of visuomotor skills (post)	Properties of the ultrasound simulator-Total score (T2b)	0.47	< 0.001
objective – subjective	Theory Test-Total score (T2b)	Properties of the ultrasound simulator-Total score (T2b)	0.30	0.01
subjective – subjective	Comfort level in performing ob/gyn TVS in-dependently (Delta)	Properties of the ultrasound simulator-Total score (T2b)	0.43	< 0.001
subjective – subjective	Comfort level in performing ob/gyn TAS independently (Delta)	Improvement in visuomotor skills (T2b)	0.41	< 0.001

subjective – subjective	Comfort level in performing ob/gyn TVS in-dependently (Delta)	Improvement in visuomotor skills (T2b)	0.48	< 0.001
objective – objective	Qualitative assessment Practical Test-Total score	Practical test-Total score	0.64	< 0.001
objective – objective	Correct interpretation of findings-Total score	Practical test-total score	0.95	< 0.001
objective – objective	Total interpretation	Qualitative assessment Practical Test-Total score	0.37	< 0.001
objective – objective	Total score deviation	Qualitative assessment Practical Test-Total score	-0.34	0.02
subjective – subjective	Comfort level in performing ob/gyn TVS independently (Delta)	Comfort level in performing ob/gyn TVS independently (Delta)	0.71	< 0.001
subjective – subjective	Advantages of simulator-based ultrasound Training-Total score (Delta)	Learning goals (Delta)	0.45	< 0.001
